# Nonlinear delay differential equations and their application to modeling biological network motifs

**DOI:** 10.1038/s41467-021-21700-8

**Published:** 2021-03-19

**Authors:** David S. Glass, Xiaofan Jin, Ingmar H. Riedel-Kruse

**Affiliations:** 1 Department of Molecular Cell Biology, Weizmann Institute of Science, Rehovot, Israel; 2 Gladstone Institutes, San Francisco, CA USA; 3 Department of Molecular and Cellular Biology, and (by courtesy) Departments of Applied Mathematics and Biomedical Engineering, University of Arizona, Tucson, AZ USA

**Keywords:** Cellular signalling networks, Differential equations, Nonlinear dynamics

## Abstract

Biological regulatory systems, such as cell signaling networks, nervous systems and ecological webs, consist of complex dynamical interactions among many components. Network motif models focus on small sub-networks to provide quantitative insight into overall behavior. However, such models often overlook time delays either inherent to biological processes or associated with multi-step interactions. Here we systematically examine explicit-delay versions of the most common network motifs via delay differential equation (DDE) models, both analytically and numerically. We find many broadly applicable results, including parameter reduction versus canonical ordinary differential equation (ODE) models, analytical relations for converting between ODE and DDE models, criteria for when delays may be ignored, a complete phase space for autoregulation, universal behaviors of feedforward loops, a unified Hill-function logic framework, and conditions for oscillations and chaos. We conclude that explicit-delay modeling simplifies the phenomenology of many biological networks and may aid in discovering new functional motifs.

## Abstract (web summary)

Network motif models focus on small sub-networks in biological systems to quantitatively describe overall behavior but they often overlook time delays. Here, the authors systematically examine the most common network motifs via delay differential equations (DDE), often leading to more concise descriptions.

## Introduction

Biological regulation consists of complex networks of dynamical interactions^1–5^. Transcription factors control production of other transcription factors^6,7^, kinases the activation of other kinases^1,3,4^, cells the growth of other cells^8,9^, and species the population size of other species^10^. “Network motifs” describe particularly important substructures in biological networks, such as negative feedback, feedforward regulation and cascades^2,6,11^. The function of network motifs often depends on emergent properties of many parameters, notably delays^7,12,13^, and previous work showed that delays can lead to dramatic biological changes, such as sustained oscillations^12,14–17^. Delays are crucial to behavior in natural^18–20^ and synthetic^21,22^ genetic oscillators, in development and disease^2,7,14,16,17,23–31^, and in ecological booms and busts^8^. Despite such examples, there has been no thorough treatment of network motifs with delays included explicitly.

**Fig. 1:**
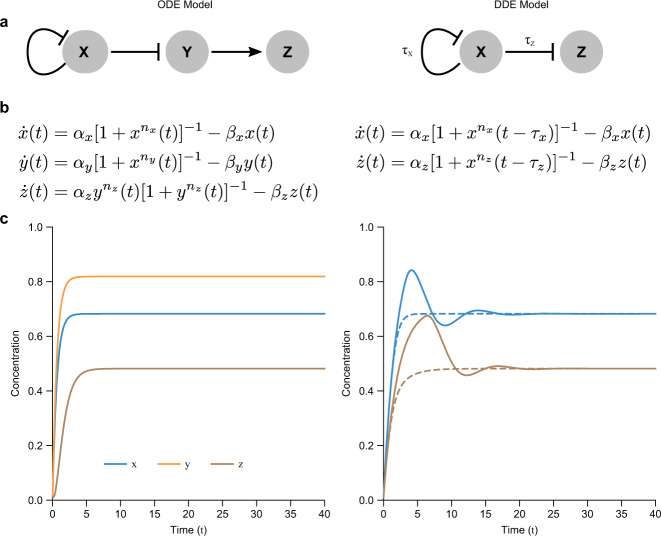
Explicit inclusion of time delays in mathematical models of network motifs can reproduce non-delay models using fewer variables and parameters, but can also lead to more complex behavior. **a** An example genetic regulatory network including genes *X*, *Y*, and *Z*, regulated by one another either positively (activation, pointed arrow) or negatively (repression, blunt arrow). The model on the left incorporates no explicit delay terms, whereas the model on the right incorporates explicit delay terms *τ*_*x*_ and *τ*_*z*_. **b** Ordinary differential equations (ODEs) and delay differential equations (DDEs) corresponding to (**a**) with regulation strengths *α*_*i*_, removal rates *β*_*i*_ and cooperativities *n*_*i*_. **c** Numerical simulations of equations in (**b**) for one set of initial conditions using parameters *α*_*x*_ = 1, *α*_*y*_ = 1.2, *α*_*z*_ = 1.2, *β*_*x*_ = *β*_*y*_ = *β*_*z*_ = 1, *n*_*x*_ = *n*_*y*_ = *n*_*z*_ = 2 for the ODE simulation, and *α*_*x*_ = 0.5, *α*_*z*_ = 0.3529, *β*_*x*_ = *β*_*z*_ = 0.5, *n*_*x*_ = *n*_*z*_ = 2 for the DDE simulations (dashed curves: *τ*_*x*_ = 0.8, *τ*_*z*_ = 0.1; solid curves: *τ*_*x*_ = 3, *τ*_*z*_ = 4). Note that delays can cause more complex dynamics (e.g., transient oscillations) compared to models in which effects are instantaneous, and where both models give the same long-term steady state behavior. Left: instantaneous effects modeled using ODEs, Right: delayed effects modeled using DDEs.

Here we provide a theoretical and practical basis for analyzing network motifs with explicit delays, and we demonstrate the utility of this approach in a variety of biological contexts. Whereas network motifs are typically modeled using ordinary differential equations (ODEs) with variables reacting to one another instantaneously (timed by rate constants), we explore network motif modeling using delay differential equations (DDEs), which have derivatives depending explicitly on the value of variables at times in the past. For example,

1 $$\dot{x}(t)=\alpha x(t)$$

is an ODE, and

2 $$\dot{x}(t)=\alpha x(t-\tau )$$

is a corresponding DDE, where *x*(*t* − *τ*) represents the value of *x* at a time *τ* units in the past, making the effect of *x* on the current rate of change of *x* delayed by a time *τ*. The time scales and delays are thus explicit (Fig. 1), better capturing dynamics where intermediate steps are not fast, as compared to ODE models, which may fail to capture real delays, hide their effects by oversimplification, or require additional variables and parameters to predict complex phenomena^17,23,32^. Such delay models have been productive in a variety of biological scenarios, such as processes with many intermediate but unimportant steps^33,34^ and age-structured populations^34,35^. Network models with delays have also been explored in various contexts^36–39^. Intercellular signal transduction, for example, requires multiple time-consuming steps that are not negligible^23,32^.

With DDEs, multiple steps (“cascades”) within a network can be rigorously simplified into a single step with delay (see below), an approach which has been explored in a variety of biological contexts^18,33,34,40–42^. This makes interpretation of the phenomenology simpler than with ODEs and reduces the number of equations and parameters in the model^18,23^. This idea can be visualized by depicting regulatory networks as directed graphs, with nodes representing biological species, and pointed vs. blunt arrows indicating activation vs. repression, respectively (Fig. 1). DDE models allow a single arrow to faithfully capture many biochemical steps^18,33,43^, expanding the available dynamics in a model with a reduced set of equations and parameters. DDE regulatory models have in fact been used widely to model a range of biological phenomena such as development^17,44^ and hematopoiesis^30,31^. For instance, a 1-variable ODE such as Eq. (1) can only produce exponential growth or decay^45,46^. A corresponding DDE, such as Eq. (2), can also oscillate (with amplitude approaching zero or infinity) and, if nonlinear, lead to stable oscillations, bistability and chaos^13,26,47–49^.

A key challenge in using DDEs is their mathematical complexity relative to ODEs^50,51^. For example, while Eq. (1) is 1-dimensional because one initial condition (*x* at *t* = 0) determines all future behavior, Eq. (2) is infinite-dimensional, because *x* must be specified at all times − *τ* ≤ *t* ≤ 0 to predict a unique solution^50,51^. Concretely, the solution to Eq. (1) can be written as a single exponential, while Eq. (2) generally must be specified as a sum of an infinite number of exponential terms to satisfy initial conditions.

Despite the challenges, much progress has been made in analytical understanding of DDEs^46,52,53^, and numerical methods exist for simulation^54,55^. We thus see an opportunity to use DDEs to recapitulate dynamics found in ODE solutions of network motif behavior with fewer genes and thus fewer modeling parameters and equations (Fig. 1), a type of “modeling simplicity.”

In this work, we thoroughly examine the most common network motifs^2,6^ with explicit delays and present an approachable, step-by-step view of the mathematical analysis (applying established DDE methods^34,51^) in order to make such delay equations easy to use for biologists and others. We show the essential role of delays in autoregulation, feedforward loops, feedback loops, multiple feedback, and complex networks, as well as instances where delays may be ignored. A reference summary is provided at the end in Table 2. Finally, we discuss how these results can be applied to understanding fundamental design principles of various natural biological systems.

## Results

Our results are divided up into 7 sections corresponding to 7 different regulatory networks of increasing complexity. An overall reference table (Table 2) is included in the discussion.

### Motif 0: direct Hill regulation

We first describe how we use DDEs to model simple regulation of a gene *Y* by a gene *X* as the most basic motif, and then provide a simple yet unified mathematical framework for both activation and inhibition with delay.

#### Activation and inhibition can both be modeled with a single unified function

**Fig. 2:**
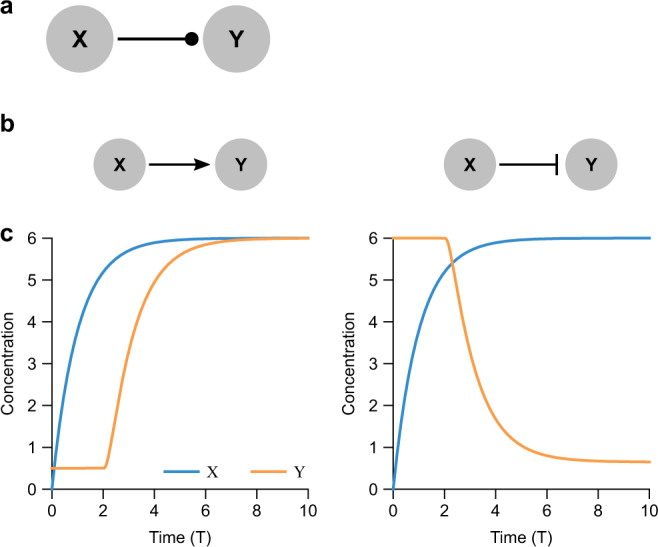
Direct regulation by activators and inhibitors can be captured using a unified delay model. **a** The simplest regulation network consists of a single input *X* directly regulating a single output *Y*. We use a dotted arrow to represent either activation or repression, with an implied explicit delay. **b** Direct activation (left) is represented by a pointed arrow and direct repression (right) by a blunt arrow. **c** Time response (Eq. (7)) of activated (left) and repressed (right) *Y* following a rise in *X* that exponentially approaches a new steady state (*η*_*X*_ = 6) from zero (governed by $\dot{X}(T)={\eta }_{X}-X(T)$). Note that the finite value of *η*_*X*_ leads to an effective leakage slightly greater than *ϵ* for the repressor case. For the activator (*n* = −2), we chose *η* = 5.6528 to match esthetically between the *X* and *Y* steady states, such that ${\eta }_{X}=\epsilon +\eta /(1+{\eta }_{X}^{n})$. For the repressor (*n* = 2), we similarly chose *η* = 5.5 to match esthetically between the *X* steady state and *Y* initial condition, such that *η*_*X*_ = *η* + *ϵ*. In both cases, *γ* = 2, *ϵ* = 0.5, and *η*_*X*_ = 6. Initial conditions were *X* = *Y* = *Z* = 0.01 for all *T* ≤ 0.

We consider a transcription factor *x* regulating the production of a protein *y* (Fig. 2). If *x* activates *y*, the production rate of *y* increases with increasing *x*, generally saturating at a maximum rate *α*. Often there is an additional cooperativity parameter *n* which determines how steeply *y* increases with *x* in the vicinity of the half-maximal *x* input value *k*. In this framework, *n* = 0 is constitutive production, *n* = 1 is a Michaelis-Menten regulation, 1 < *n* < 5 is a typical biological range of cooperativity, and *n* → *∞* is a step-function regulation. If *x* represses *y*, the same conditions hold except that the production rate of *y* then decreases with increasing *x*. A standard quantitative model for this behavior is called the Hill function, and serves as a good approximation for many regulatory phenotypes in biology, including transcription and translation rates, phosphorylation, enzymatic activity, neuronal firing, and so forth^56,57^. In general there may also be a leakage rate *α*_0_ that yields constant *y* production in the absence of *x*. The concentration of *y* is also generally removed at a rate *β* proportional to its concentration. This removal term can represent many biophysical processes, such as degradation, dilution, compartmentalization, or sequestration^58,59^; for simplicity we mainly use the term “degradation.”

Together these can be written as:

3 $$\dot{y}(t)={\alpha }_{0}+\frac{\alpha {x}^{n}(t)}{{k}^{n}+{x}^{n}(t)}-\beta y(t)\qquad {\rm{(activator)}}.$$

If *x* instead “represses” *y* instead of activating it, the form is similar:

4 $$\dot{y}(t)={\alpha }_{0}+\frac{\alpha {k}^{n}}{{k}^{n}+{x}^{n}(t)}-\beta y(t)\qquad {\rm{(repressor)}}.$$

For biologically meaningful results, all variables and parameters in these equations should be real and non-negative. Note, however, that the activator case is equivalent to the repressor case with *n* < 0. In this paper we will therefore allow *n* to be negative, which is simply a notational modification used in order to combine the two cases. With this notation, the effective cooperativity is ∣*n*∣. Thus, we have:

5 $$\dot{y}(t)={\alpha }_{0}+\frac{\alpha {k}^{n}}{{k}^{n}+{x}^{n}(t)}-\beta y(t)\quad \left\{\begin{array}{ll}-\infty \,<\,n\,<\,0\hfill&\!\!\!\!\!\!\!({\rm{activator}})\\ n=0\hfill&({\rm{constitutive}})\\ 0\,<\,n\,<\,\infty\hfill &\!\!\!\!\!\!({\rm{repressor}})\end{array}\right..$$

which provides a unified, single-function description for both activators and inhibitors (and constitutive expression), providing a powerful mechanism to analyze both cases simultaneously as we will do throughout this work.

The negative value of *n* allowed here should not be confused with the term “negative cooperativity” used in the binding kinetics literature^60^, which in our notation would refer strictly to a negative value of *n* with magnitude less than one (i.e., − 1 < *n* < 0). The Hill function has an inflection point for all ∣*n*∣ > 1, which allows there to be 3 fixed points for *n* < 0 when feedback is introduced below (Supplementary Fig. 1).

#### Delays may be present in regulation, but are not modeled in removal

Converting Eq. (5) from ODE to DDE to account for explicit delay *τ* in regulation, we replace *x*(*t*) with *x*(*t* − *τ*) in the regulation (Hill) term, but not in the removal term, which is directly dependent on *y* itself, and thus not expected to have any delay. A delayed removal term can also in general lead to negative values, limiting its use in a biological context. This form of regulation is quite general, but for concreteness we will generally refer to quantities like *x* (or *y*) as the concentrations of some protein *x*. Thus we arrive at an explicit-delay DDE model of activating or repressive biological regulation as follows:

6 $$\dot{y}(t)={\alpha }_{0}+\frac{\alpha {k}^{n}}{{k}^{n}+{x}^{n}(t-\tau )}-\beta y(t)\quad \left\{\begin{array}{ll}-\infty \,<\,n\,<\,0\hfill&({\rm{activator}})\\ n=0\hfill&\,\,\,\,\,\,\,({\rm{constitutive}})\\ 0\,<\,n\,<\,\infty \hfill&\,({\rm{repressor}})\end{array}\right..$$

#### Nondimensionalizing yields 4 key parameters for any regulation

We can non-dimensionalize Eq. (6) by dividing all concentrations by the half-maximal input concentration *k* and dividing times by the degradation time 1/*β*, which has the effect of measuring concentrations in units of *k* and times in units of 1/*β*. This yields:

7 $$\dot{Y}(T)=\epsilon +\frac{\eta }{1+{X}^{n}(T-\gamma )}-Y(T)\quad \left\{\begin{array}{ll}-\infty \,<\,n\,<\,0\hfill&({\rm{activator}})\\ n=0\hfill&\,\,\,\,\,\,\,({\rm{constitutive}})\\ 0\,<\,n\,<\,\infty \hfill&\,({\rm{repressor}})\end{array}\right.,$$

with dimensionless variables *X* = *x*/*k*, *Y* = *y*/*k*, *T* = *tβ*, and dimensionless parameters *ϵ* = *α*_0_/*kβ*, *η* = *α*/*kβ*, *γ* = *τβ*, and *n*. We thus reduce the number of parameters from 6 to 4, and as discussed below, primarily *η* and *γ* are important. In this form, *η* is a normalized “regulation strength” and *γ* is a normalized delay. These dimensionless parameters can be considered “big” or “small” by comparing their value relative to unity. For example, *γ* = *τβ* ~ 1 implies that the delay (*τ*) and degradation (1/*β*) times are approximately equal. In subsequent sections we generally explore parameter values between 0.1 and 10. The dynamics of direct activation and repression via Eq. (7) are demonstrated by numerical simulation in Fig. 2c. For simplicity, here and in all other simulations, we use constant initial conditions (variables constant in time for *T* ≤ 0) unless otherwise specified.

### Motif I: cascade (sequential regulation)

**Fig. 3:**
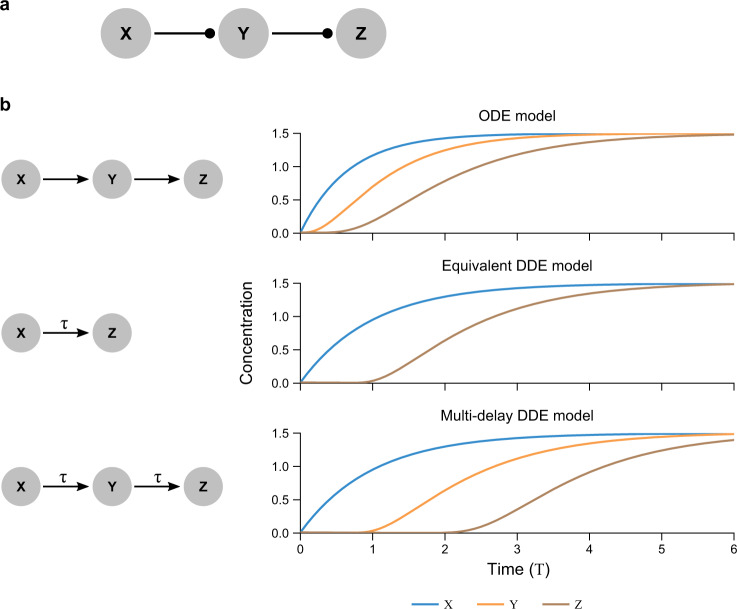
A delayed model of cascades recapitulates the implicit delays in ODE models. **a** A cascade is a linear sequence of regulation steps, here *X* regulating *Y* regulating *Z*. **b** Standard models of cascades use ODEs (top), in which each step leads to a characteristic delay in the products *Y* and *Z*, which increases with each step based on the half-maximal inputs and degradation rates for each step. In an equivalent DDE model (middle), similar behavior is accomplished by replacing the explicit cascade of implicit delays with a single-step regulation including an explicit delay. A cascade in which each step contains an explicit delay (bottom) behaves analogously to delayed direct regulation (as in middle), with the final step delayed by the sum of delays in each step. *η*_*X*_ = 1.5, *η*_*Y*_ = *η*_*Z*_ = 2.17, *β*_*X*_ = *β*_*Y*_ = 1, *β*_*Z*_ = 0.667, *n*_*X*_ = *n*_*Y*_ = − 2. For the bottom graph in (**b**), each step is governed by Eq. (9) with identical parameters.

A common network motif in many biological networks is the cascade (Fig. 3), a series of regulatory steps (i.e., *x* regulates *y*, which regulates *z*, etc.)^2,6,61^. Since each regulator must reach the corresponding half-maximal input value *k* before significantly affecting the next item in the cascade, each step adds an effective delay. The following analysis falls within a range of methods used to replace intermediate steps with a delay^18,41,42^.

#### Delayed direct regulation approximates cascades of non-delayed regulation

For a non-delayed cascade motif in which *X* regulates *Y*, which in turn regulates *Z* (Fig. 3), one can write down a nondimensional set of governing equations as follows:

8 $$\frac{{\beta }_{z}}{{\beta }_{x}}\dot{X}(T)	= \;{\eta }_{X}-X(T)\\ \frac{{\beta }_{z}}{{\beta }_{y}}\dot{Y}(T)	= \;\frac{{\eta }_{Y}}{1+{X}^{{n}_{X}}(T)}-Y(T)\\ \dot{Z}(T)	= \;\frac{{\eta }_{Z}}{1+{Y}^{{n}_{Y}}(T)}-Z(T),$$

in which *β*_*x*_, *β*_*y*_, and *β*_*z*_ are the dimensional degradation rates of *X*, *Y*, and *Z*, respectively (Supplementary Note 1). We have included here an explicit input function *X* for concreteness, but more generally we can consider any input *X*(*T*) with characteristic timescale 1/*β*_*x*_.

If *Y* changes quickly compared to *X* (i.e., *β*_*x*_/*β*_*y*_ ≪ 1), a first approximation to arriving at a simplified model that ignores *Y* is to replace the appearance in $\dot{Z}(T)$ of *Y*(*T*) with the *X*-dependent pseudo-steady state *Y*_*pss*_(*X*(*T*)) found by setting $\dot{Y}(T)=0$. A more precise estimate with the looser demand that ${({\beta }_{x}/{\beta }_{y})}^{2}\ll 1$ can be found by using the same pseudo-steady state, but delayed in time as *Y*_*pss*_(*X*(*T* − *γ*)), with *γ* = *β*_*z*_/*β*_*y*_ (Supplementary Note 1). Intuitively, this delay takes into account the fact that on the slower timescale of *X*, *Y* effectively follows the dynamics of *X*, but the delay accounts for the non-zero time *β*_*z*_/*β*_*y*_ that it takes for *Y* to respond to such changes in *X* (the *β*_*z*_ deriving merely from normalizing time *T* = *tβ*_*z*_). By removing *Y* we lose certain information, such as the ratio of *Z* or *X* to *Y*, but the inclusion of a delay provides for a reasonably quantitative approximation of the output *Z*’s dynamics as a function of the input *X*.

After plugging the delayed pseudo-steady state of *Y* into $\dot{Z}(T)$, one can match the values of the composite Hill-within-Hill function at *X* = 0, *X* = 1, and *X* → *∞* as well its the slope at *X* = 1 compared to a single Hill function with leakage, and thereby approximate the cascade as a single-step regulation of *Z* by *X* (Supplementary Note 1):

9 $$\dot{Z}(T)\approx {\alpha }_{0}+\frac{\alpha {k}^{h}}{{k}^{h}+{X}^{h}(T-\gamma )}-Z(T).$$

where the combined regulation parameters are (Supplementary Note 1)

10 $$\begin{array}{lll}{\alpha }_{0}=\frac{{\eta }_{Z}}{1+{\eta }_{Y}^{{n}_{Y}}}\left(\frac{{\mathrm{sgn}}\,{n}_{Y}+1}{2}\right)\hfill& \alpha =\frac{{\eta }_{Z}{\eta }_{Y}^{| {n}_{Y}| }}{1+{\eta }_{Y}^{| {n}_{Y}| }}\hfill\\ {k}^{h}={\left(\frac{{2}^{| {n}_{Y}| }-1}{1+{\eta }_{Y}^{| {n}_{Y}| }}\right)}^{{\mathrm{sgn}}\,{n}_{Y}}& h=-\frac{{n}_{X}{n}_{Y}}{2}\left(\frac{{2}^{| {n}_{Y}| }}{{2}^{| {n}_{Y}| }-1}\right)\end{array}\quad .$$

The *k* can be removed by renormalization of *X*.

Equation (10) has several biological consequences. First, the overall Hill coefficient *h* is negatively proportional to the product of individual cooperativities *n*_*X*_, *n*_*Y*_. This makes the cascade activating for either two activators or two repressors, and repressive otherwise. It also makes the cascade more cooperative than its components, in line with past analyses^62^ and as found experimentally^61^. Third, the leakage is zero for *n*_*Y*_ < 0 and negligible (i.e., *α*_0_ ≪ *α*) for *n*_*Y*_ > 0 if *η*_*Y*_ ≫ 1. This means *Y* must be produced in sufficient amount during the delay to repress *Z*. We generally ignore leakage in the main text; for a more detailed discussion see Supplementary Note 3 and Supplementary Fig. 2.

#### The total delay in a cascade equals the sum of individual delays

For cascades with delays at each step, the same analysis implies that total delay equals the sum of individual delays (*β*_*z*_/*β*_*y*_ + *γ*_*y*_ + *γ*_*z*_, with *γ*_*y*_ the intrinsic delay between *X* and *Y* and *γ*_*z*_ the intrinsic delay between *Y* and *Z*). Figure 3 shows simulations of ODE, equivalent DDE, and multi-delay DDE cascade models, matching the above analytical results.

### Motif II: autoregulation

**Fig. 4:**
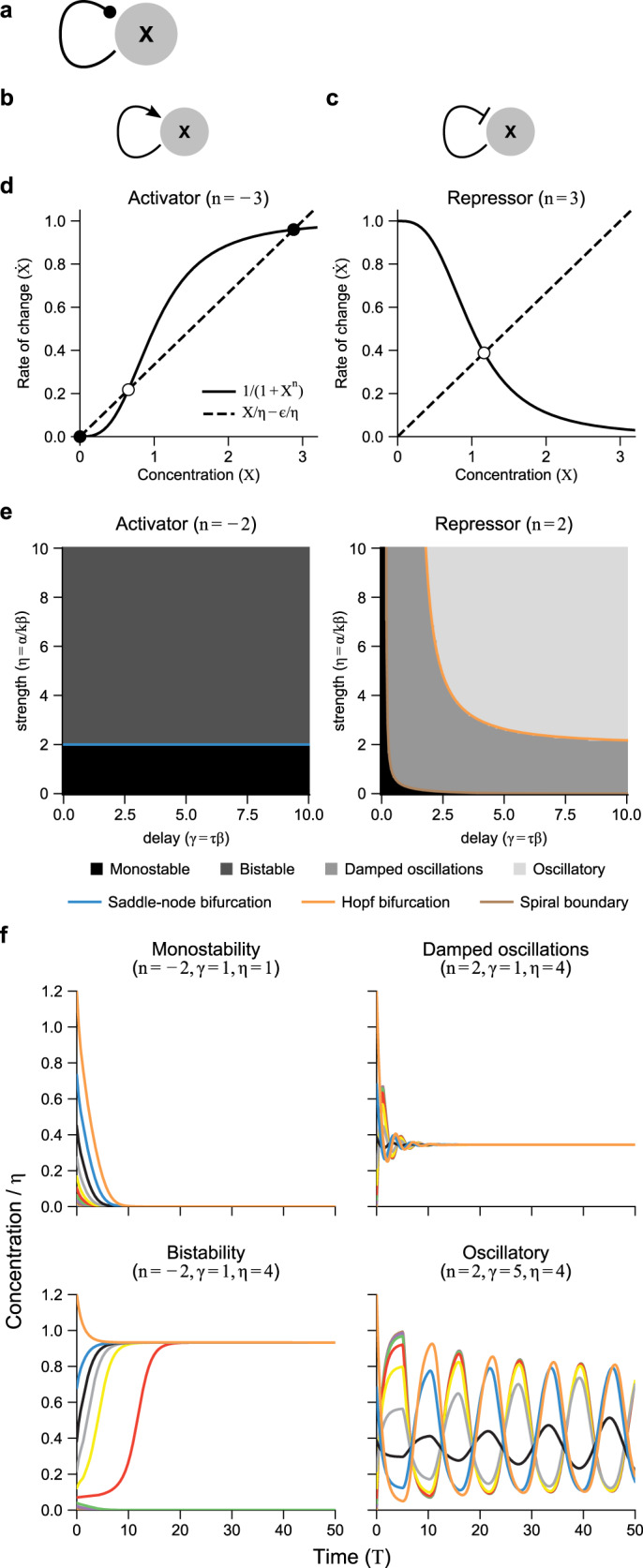
The complete phase diagram for the autoregulation network motif has analytically derivable parameter regions for bistability, monostablility, monostablility with damped oscillations, and oscillations. **a** The autoregulation network motif, with a dotted arrow indicating either (**b**) self-activation or (**c**) self-repression. The two cases are given by *n* < 0 (activation) and *n* > 0 (repression). **d** Stable (black circles) and unstable (white circles) fixed points for activator and repressor cases are given by the intersection of the regulation (solid) and degradation+leakage (dashed) lines. Note that activators can have 1, 2, or 3 fixed points, whereas repressors always just have one. **e** Parameter space showing all possible behaviors for autoregulation with delay. Shading shows simulation results (with an interval of 0.1 for both *γ* and *η* axes) and curves show the analytically derived bifurcation boundaries. See Supplementary Fig. 3a for cases −3 ≤ *n* ≤ 3 and Supplementary Fig. 3b for boundaries in *η* vs. *n* parameter space. **f** Representative simulation curves for the four qualitatively different behaviors, with different colors representing different initial conditions (see “Methods”). *ϵ* = 0.

Autoregulation, one of the most common biological motifs^2,63^, describes a single biological species that regulates its own production (Figs. 4a–c).

#### The complete phase space for autoregulation demonstrates the quantitative and qualitative importance of delays

Based on Eq. (5), the governing equation for such a system with delayed regulation is given by setting *Y* = *X* (output equals input) in Eq. (7):

11 $$\dot{X}(T)=\epsilon +\frac{\eta }{1+{X}^{n}(T-\gamma )}-X(T).$$

This equation has four parameters (*η*, *γ*, *ϵ*, and *n*).

Since leakage must be small relative to regulation (i.e., *ϵ*/*η* ≪ 1) for regulation to be strongly effective (“activated” rate much greater than “non-activated” rate), we will focus here on the case with no leakage (*ϵ* = 0). We treat non-negligible leakage in the supplements (Supplementary Note 3). Note that in general Eq. (11) has no closed-form solution.

#### Fixed points do not depend on delays

Fixed points *X*(*T*) = X^*^ do not change with time, implying $\dot{X}(T)=0$ and *X*(*T*) = *X*(*T* − *γ*) = X^*^. In Eq. (11), this implies that production must balance degradation (Fig. 4d). For repressors (*n* > 0) there is only a single fixed point for biologically relevant parameter values. For activators (*n* < 0), there can be 1, 2, or 3 fixed points (2 fixed points is a border case for −*n* > 1). Explicitly, the fixed point values are given by

12 $${X}^{* }\left(1+{{X}^{* }}^{n}\right)=\eta$$

or X^*^ = 0 (for activators only). Again, we assumed *ϵ* = 0 for simplicity. Note that these fixed points only depend on 1/*η* = *βk*/*α* and *ϵ*/*η* = *α*_0_/*α*, and have no dependence on the delay time *τ*.

#### Linearization is sufficient to determine bifurcations in qualitative behavior

Small disturbances from fixed points *δX*(*T*) = *X*(*T*) − X^*^ ≪ 1 linearize Eq. (11) (Supplementary Note 2). Assuming a solution $\delta X(T)=A\exp (\lambda T)$ yields a transcendental “characteristic equation” for the eigenvalues *λ* of Eq. (11):

13 $$\lambda +\eta M{e}^{-\gamma \lambda }+1=0.$$

The constant function of the fixed point value *M*(X^*^) is defined as

14 $$M({X}^{* })=\frac{n{{X}^{* }}^{n-1}}{{\left(1+{{X}^{* }}^{n}\right)}^{2}},$$

whose sign is given by *n* (positive for repressors and negative for activators).

To determine stability of the fixed points, we solve for conditions in which *λ* crosses the imaginary axis ($\mathrm{Re}\,\lambda =0$), occuring at either *λ* = 0 (a saddle-node bifurcation) or *λ* = *iω* (a Hopf bifurcation).

#### Saddle-node bifurcations determine bistability in autoactivators

For the saddle-node case (*λ* = 0), Eqs. (13) and (12) reduce to (Supplementary Note 2):

15 $$\eta =-n{(-n-1)}^{-\frac{-n-1}{-n}}.$$

which is biologically meaningful ($\eta \,> \, 0,\mathrm{Im}\,\eta =0$) only for autoactivation (*n* < 0), where − *n* is the biologically relevant quantity. We can see that Eq. (15) separates monostability from bistability by noting that the production and removal curves (Fig. 4d) are tangent when Eq. (15) holds (Supplementary Note 2), yielding two stable fixed points when *η* is above this boundary and only one otherwise. For −1 < *n* ≤ 0, the origin (X^*^ = 0) is unstable (since *M*(X^*^ = 0) → −*∞*), and it ceases to be a fixed point for *n* > 0. Note that this boundary does not depend on delay; however, that does not preclude other features such as basins of attraction from depending on delay^64^.

Viewed as a function of *n*, Eq. (15) limits bistability to −*n* > 1 and the boundary to 1 ≤ *η* ≤ 2 (Supplementary Fig. 1b). Since this function is non-monotonic, it is also possible (although biologically perhaps unrealistic except on an evolutionary timescale), to hold a value of 1 < *η* < 2 and decrease *n* to a point where the bistability is lost, and then decrease *n* still further until bistability is gained again.

#### Hopf bifurcations determine oscillatory behavior in autorepressors

For the Hopf bifurcation (*λ* = *iω*), Eqs. (13) and (12) result in two equations, one for the real parts of Eq. (13) and one for the imaginary parts (Supplementary Note 2):

16 $$\begin{array}{ll}\gamma =\frac{1}{\omega }\left(-{\tan }^{-1}\omega +\pi k\right),\qquad k=0,1,\ldots \\ \eta =\frac{| n| }{\sqrt{1+{\omega }^{2}}}{\left(\frac{| n| }{\sqrt{1+{\omega }^{2}}}-1\right)}^{-\frac{n+1}{n}}\; .\hfill\end{array}$$

Equation (16) represent a series of curves parameterized by *ω*, and is biologically meaningful (*γ* > 0, *η* > 0) for *k* ≥ 1. For autoactivation (*n* < 0), these curves all lie above the bistability boundary (Eq. (15)), and thus have no qualitative effect on behavior. For autorepression (*n* > 0), however, the outermost curve (*k* = 1) dictates the onset of oscillations for any *n* > 1.

This boundary has both horizontal (*η*) and vertical (*γ*) asymptotes, given as:

17 $$\lim_{\omega \to 0}{\eta }_{{\rm{Hopf}}}= \;| n| {(| n| -1)}^{-\frac{n+1}{n}}\\ \lim_{\omega \to \sqrt{{n}^{2}-1}}{\gamma }_{{\rm{Hopf}}}= \;\frac{{\cos }^{-1}\left(-| n{| }^{-1}\right)}{\sqrt{{n}^{2}-1}}.$$

Thus oscillations require both a minimal regulation strength and a minimum delay. The vertical (*γ*) asymptote approaches zero as *n* → *∞*, so large regulatory strength can achieve oscillations even with minuscule delay if cooperativity is extremely steep.

The oscillation period can be approximated by linearization near consecutive maxima and minima of the oscillation (Supplementary Note 2). This method yields a period of approximately 2(*γ* + 1), or 2(*τ* + 1/*β*) in dimensionful terms (compare also to^17^). Biologically, this means that the concentration *X* is pushed from high to low (or vice versa) after the delay time (*τ*) has elapsed and the concentration has equilibrated to its new value via degradation (1/*β*).

Damped oscillations are expected when the largest eigenvalue satisfying Eq. (13) has non-zero imaginary part. This is never true for *n* < 0, and is true for *n* > 0 above the curve denoted “spiral boundary” in Fig. 1e and Supplementary Fig. 3. This curve is given as follows:

18 $$\eta =n\gamma {e}^{\gamma +1}{\left(n\gamma {e}^{\gamma +1}-1\right)}^{-\frac{n+1}{n}},$$

which is derived in Supplementary Note 4. It approaches *η* = 0 for large delay and has a vertical asymptote at *nγe*^*γ*+1^ = 1, so there is some non-zero delay that has non-oscillating decay for any strength *η* and finite *n*.

Putting together the derived boundary curves yields an *η*-*γ* phase space for each *n*, displayed in Fig. 4e and Supplementary Fig. 3a-b along with simulations showing that this analytical treatment matches the dynamical behavior.

#### Leakage prevents oscillations and bistability

Autoregulation with leakage (Supplementary Note 3) adds the extra parameter *ϵ* ≠ 0. Small leakage *ϵ* ≪ *η* does not change the qualitative results while large leakage effectively overwhelms the regulation term, preventing both oscillations (for autorepression) and bistability (for autoactivation). The full treatment with leakage and the corresponding phase portraits are presented in the supplements (Supplementary Note 3, Supplementary Fig. 2).

### Motif III: logic

**Fig. 5:**
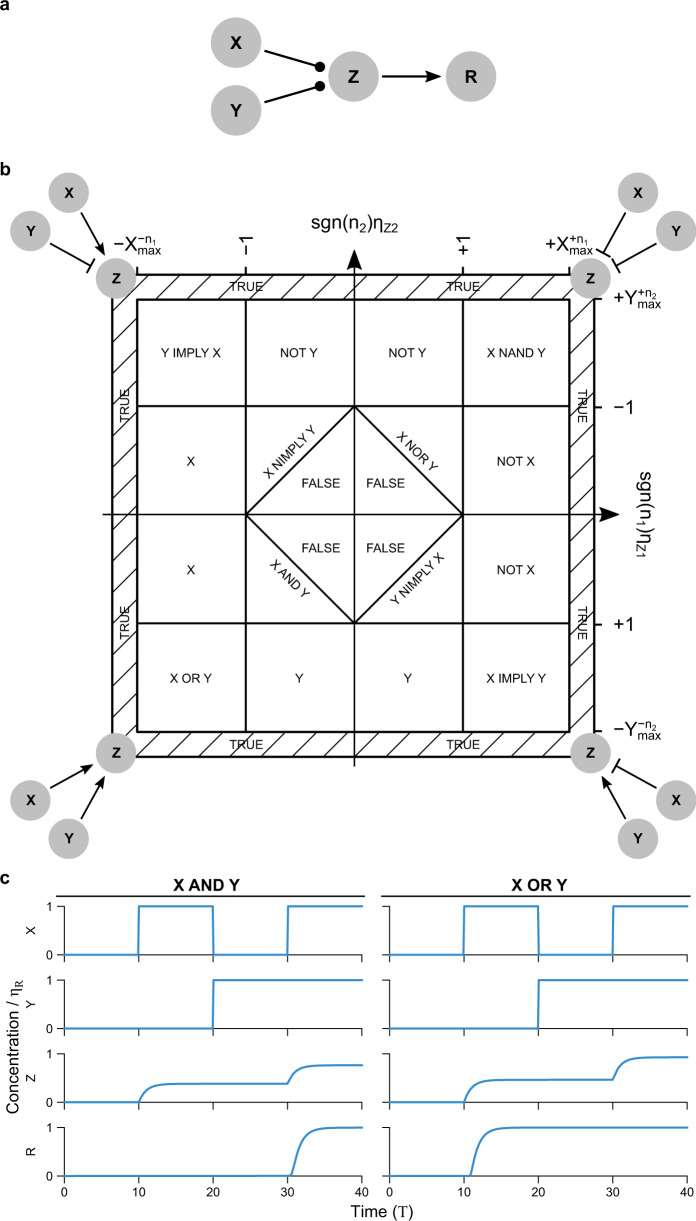
A simple approximation for digital logic using a sum of Hill terms recapitulates all monotonic logic functions in a single parameter space. **a** A prototypical regulatory network involving logic where X and Y both regulate Z, which must integrate the two signals using some logic before it can in turn activate a downstream reporter R. **b** Parameter space showing regions where regulation approximately follows 14 of the 16 possible 2-input logic functions depending on the strength of two single-variable Hill regulation terms (*η*_*Z*1_: regulation of Z by X, *η*_*Z*2_: regulation of Z by Y). Network logic can be smoothly altered by varying the parameters (*η*_*Z*1_, *η*_*Z*2_), with a change of sign in (*n*_1_, *n*_2_) required to switch quadrants. The bottom-left quadrant shows that very weak regulation in both terms leads to an always-off (FALSE) function, weak regulation in one arm only leads to single-input (X, Y) functions, strong regulation in both arms leads to an OR function, and regulation too weak in either arm alone to activate an output but strong enough in sum leads to an AND function. The other three quadrants are related by applying NOT to one or both inputs, with function names related by de Morgan’s law^70^ NOT(X OR Y) = NOT X AND NOT Y. In particular, X IMPLY Y = NOT(X) OR Y, X NIMPLY Y = X AND NOT(Y), X NOR Y = NOT X AND NOT Y, and X NAND Y = NOT X OR NOT Y. Truth tables for all 16 logic gates are provided in Supplementary Table 1 for reference. The two non-monotonic logic functions, X XOR Y and X XNOR Y, are those 2 of 16 not reproduced directly using this summing approximation. They can be produced by layering, e.g., NAND gates^70^. **c** Representative time traces for AND (*η*_*Z*1_ = *η*_*Z*2_ = 0.9) and OR (*η*_*Z*1_ = *η*_*Z*2_ = 1.8) gates with *n*_1_ = *n*_2_ = −2, *n*_3_ = −20, *η*_*R*_ = *η*_*Z*1_ + *η*_*Z*2_. The function $\mathrm{sgn}\,(n)=+1$ when *n* > 0, $\mathrm{sgn}\,(n)=-1$ when *n* < 0.

A general class of functions used to describe natural^2,58,65–67^ and synthetic^68,69^ biological networks are logic gates, which have two inputs regulating a single output (Fig. 5). Gates exhibit either high (“on”) or low (“off”) output depending on whether inputs are on or off. For example, the AND gate specifies high output only if both inputs are on. In this section we provide a specific DDE-based framework that covers 14 out of 16 possible 2-input logic operations, and show that these operations form a continuous 2D parameter space.

#### A two-parameter summing function reproduces all 2-input monotonic logic gates

We first write out nondimensionalized equations corresponding to the logic gate motif as depicted in Fig. 5a. We assume that the degradation constants (*β*) for *Z* and *R* are equal for simplicity, and that there is no leakage.

19 $$\begin{array}{lll}\dot{Z}(T)=\frac{{\eta }_{Z1}}{1+{X}^{{n}_{1}}(T-{\gamma }_{1})}+\frac{{\eta }_{Z2}}{1+{Y}^{{n}_{2}}(T-{\gamma }_{2})}-Z(T)\\ \dot{R}(T)=\frac{{\eta }_{R}}{1+{Z}^{{n}_{3}}(T-{\gamma }_{Z})}-R(T),\hfill\end{array}$$

We describe the regulation of *Z* by *X* and *Y* using a sum of Hill terms. Logic gate behavior can be captured with other approaches such as a product of Hill terms, or summation within a single Hill term^58,66,67,71^, each representing subtly different biology. We choose the separate Hill term approach as it describes many logic functions simply by tuning regulatory strengths, and can be extended to include multiplicative terms (Supplementary Note 5). Caveats include multiple states for *Z*, requiring additional binarization via *R* (Fig. 5c), as well as poor response to ratiometric inputs, discussed in the next section on feedforward motifs.

Using Eq. (19), we can characterize the motif logic based on the idea of dynamic range matching^72^. Every regulator in Eq. (19) is effectively compared against unity in the denominator of the Hill function for its corresponding output. For instance, *Z* provides an “on” or “off” signal to *R* if *Z* > 1 or *Z* < 1 respectively. *Z* can take on values below 1 as long as ${\eta }_{Z1}\ll \max ({X}^{| {n}_{1}| })$ and ${\eta }_{Z2}\ll \max ({Y}^{| {n}_{2}| })$, otherwise *Z* will always activate *R*, as indicated by the areas marked TRUE in Fig. 5.

Let us say that *X* and *Y* settle on steady-state values X^*^ ≡ *η*_*X*_, Y^*^ ≡ *η*_*Y*_, as inputs to our logic gate. A value of *η*_*X*_ or *η*_*Y*_ significantly greater than 1 is then “high” (true, 1), and “low” (false, 0) if much less than 1. We then want to determine whether Z^*^ is greater than (true) or less than (false) 1. For example, if *n*_1,2_ > 0 (two repressors), Table 1 gives the possible steady states of *Z*. If *η*_*Z*1_ and *η*_*Z*2_ are greater than 1, these steady states approximate a NAND gate. If they are less than 1, but sum to greater than 1, the steady states instead approximate a NOR gate.

**Table 1:** NAND Logic function for two repressors based on Eq. (19).

*η*_*X*_	*η*_*Y*_	Z^*^	Z^*^ for *η*_*Z*1,2_ > 1
≪ 1	≪ 1	*η*_*Z*1_ + *η*_*Z*2_	>1
≪ 1	≫ 1	${\eta }_{Z1}+{\eta }_{Z2}/{\eta }_{Y}^{{n}_{2}}$	>1
≫ 1	≪ 1	${\eta }_{Z1}/{\eta }_{X}^{{n}_{1}}+{\eta }_{Z2}$	>1
≫ 1	≫ 1	0	<1

Equivalent calculations for all variations of *n*_1_, *n*_2_, *η*_*X*_, *η*_*Y*_, *η*_*Z*1_, and *η*_*Z*2_ (Supplementary Table 2) show that logic is dependent on only two parameters, ${\mathrm{sgn}}\,({n}_{1}){\eta }_{Z1}$ and ${\mathrm{sgn}}\,({n}_{2}){\eta }_{Z2}$. These form a 2D parameter space that summarize the logic into a convenient chart (Fig. 5b). This implies evolution or other tuning can smoothly convert between logic gates (see also^66,67^). The chart is symmetric on interchange of the two inputs (*η*_1_ ↔ *η*_2_, *n*_1_ ↔ *n*_2_), and anti-symmetric (that is, application of logical NOT) by conversion of an activator to a repressor or vice versa (*n*_*i*_ → −*n*_*i*_). Fourteen of the 16 possible two-input logic functions are represented. All fourteen are monotonic in that *Z*(*X*, *Y* = 1) > *Z*(*X*, *Y* = 0) and *Z*(*X* = 1, *Y*) > *Z*(*X* = 0, *Y*). The two non-monotonic gates, XOR and XNOR, are not represented by this simple logic motif, because summation (addition of Hill terms) is monotonic. However, they can be constructed by connecting several of these gates^70,72^.

#### The logic parameter space can be divided into AND-type, OR-type, and single-input functions

Only the 8 regulatory functions on the diagonals of Fig. 5 (excluding FALSE and TRUE) make use of both inputs. These eight can be further divided into positive or negative regulation on each arm (4 possibilities) in conjunction with an AND or OR gate (8 possibilities total). Specifically, “OR-type” logic applies when both *η*_*Z*__1_ > 1 and *η*_*Z*__2_ > 1 (OR, NAND, and both IMPLY gates), and “AND-type” logic applies when both *η*_*Z*__1_ < 1 and *η*_*Z*__2_ < 1 (AND, NOR, and both NIMPLY gates). This can be seen mathematically by using Boolean logic simplification. For example, *X* NOR *Y* = NOT *X *AND NOT *Y*. Similarly, *X* NAND *Y* = NOT *X* OR NOT *Y*.

It is important to note that this logic scheme is an approximation, and in reality the sum of two Hill terms provides a form of fuzzy logic^73^. That is, if the inputs *X* or *Y* are close to 1, or if the regulatory strengths *η*_*Z*1,2_ are close to 1, then *Z* will also be close to 1 for some input combinations (Supplementary Note 5).

Note that if *X*(*T*) and *Y*(*T*) are independent, they can be time-shifted in Eq. (19) by *γ*_1_ and *γ*_2_, respectively, showing that the dynamics do not depend on delays. This is not true if *X* and *Y* are dependent (e.g., *X* = *Y*), which leads to interesting dynamics that we examine below in feedforward loops and double feedback.

### Motif IV: feedforward loop

**Fig. 6:**
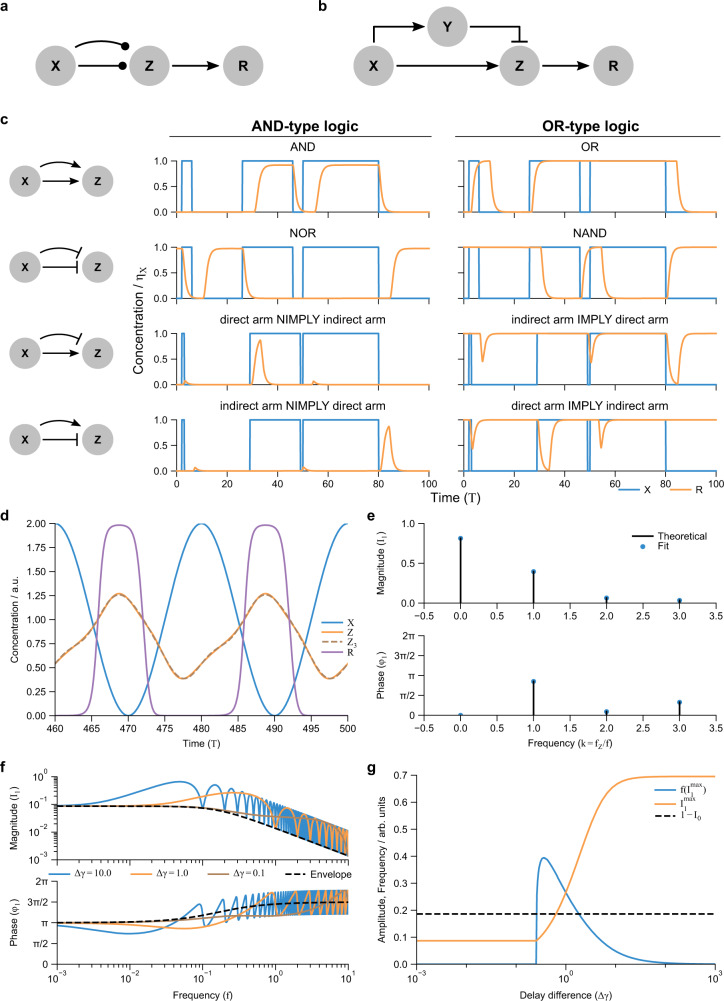
The feedforward network motif owes its primary functions to a difference in regulatory delays. **a** The feedforward motif with delays, in which a single output *Z* is controlled by an input *X* via two regulatory arms with differing delays. The straight, short arrow represents the “direct arm” with delay *γ*_1_ and the longer, curved arrow represents the “indirect arm” with delay *γ*_2_ > *γ*_1_. **b** The ODE model for an incoherent (type 1) feedforward motif, one of 8 possible networks in which the intermediate gene *Y* is modeled explicitly in the indirect arm. **c** Simulations of the four feedforward motifs with AND-type and OR-type logic (Fig. 5 and Supplementary Table 1) in response to short and long gain and loss of input signal. Blue curves: inputs (*X*), orange curves: reporter *R* activated with high cooperativity by *Z*. Note that the bottom two rows demonstrate pulse generation, while the top two rows filter short signals. **d** Response of an incoherent feedforward motif to oscillatory input after initial transients have died away. *Z*_3_ is a 3-frequency Fourier approximation of *Z* (see E). **e** Fourier decomposition of *Z* from (**d**) by Eq. (26) and by a numerical fit to the data in (**d**). **f** Frequency scan (Bode plot) of (**d**) for 3 values of Δ*γ*, with the theoretical envelopes from Eq. (28). **g** The maximum amplitude of the motif in (**d**) over a range of Δ*γ* and the corresponding frequencies at which the maxima occur. *Z* goes above 1 (activation threshold for *R*) for a small range of Δ*γ*. For (**c**–**f**), *η*_1_ = 0.9, *η*_2_ = 0.7, *n*_1_ = 2, *n*_2_ = −2, *n*_3_ = −20, *η*_*R*_ = 2, *A* = 1. For **d**, *f* = 0.05, Δ*γ* = 4.

Equipped with a multivariable generalization of 1-variable Hill regulation (Eq. (19)), we turn our attention to the feedforward motif (Fig. 6), a non-cyclic regulation network in which an input, *X*, regulates an output, *Z*, via two separate regulation arms. One arm is “direct”, in which *X* regulates *Z* in a single step, while the second arm is “indirect”, with *X* regulating an intermediate *Y*, which in turn regulates *Z*. In this way, the first arm “feeds forward” past the cascade (Fig. 6a–b). The motif is found commonly in biological networks^6,74^, comprising about 30% of three-gene regulatory interactions in transcriptional circuits^75^. Feedforward loops are conventionally described as “incoherent” if *X* activates *Z* through one arm but represses through the other, and “coherent” otherwise (Supplementary Table 3). In this section, we show that the essential behaviors of feedforward loops are due to a difference in delays between the two inputs to *Z* and the logic function between the two inputs to the output *Z*.

#### Feedforward dynamics depend only on the difference in time delay between two regulatory arms and their 2-input logic

With a DDE model, we can drop the cascaded intermediate *Y*, focusing solely on *Z* as regulated by *X* via two arms that have unequal delays (Fig. 6a, b). Looking at Fig. 6a, we can then write down a simple equation for the regulation of *Z*, by setting *Y* = *X* in Eq. (19) (Supplementary Note 6):

20 $$\dot{Z}(T)=\frac{{\eta }_{1}}{1+{X}^{{n}_{1}}(T-{\gamma }_{1})}+\frac{{\eta }_{2}}{1+{K}^{{n}_{2}}{X}^{{n}_{2}}(T-{\gamma }_{2})}-Z(T).$$

Because each regulation term is controlled by the same input (*X*), we cannot in general normalize the half-maximal input to 1 in both terms as we did for logic (Eq. (19)); *K* = *k*_1_/*k*_2_ represents the ratio of the two input scales. Likewise, we cannot entirely time-shift out the delays; instead, shifting *X* backwards in time by *γ*_1_ ($\hat{X}(T)=X(T+{\gamma }_{1})$), we see that the behavior of the feedforward equation actually depends only on the difference in regulatory delays, Δ*γ* = *γ*_2_ − *γ*_1_ rather than each delay independently:

21 $$\dot{Z}(T)=\frac{{\eta }_{1}}{1+{\hat{X}}^{{n}_{1}}(T)}+\frac{{\eta }_{2}}{1+{K}^{{n}_{2}}{\hat{X}}^{{n}_{2}}(T-{{\Delta }}\gamma )}-Z(T).$$

For clarity we will from now on refer to $\hat{X}$ as *X* without the hat unless specified.

To understand the behavior of Eq. (21), we will analyze the output of *Z* to two types of input *X*: a step input, and a continuous oscillation. Termed the step and frequency responses, respectively, these are often used in control theory to characterize input-output relations^76^.

#### Step-pulse response of feedforward motifs can be solved analytically and demonstrates filtering and pulse-generation behaviors

We considered a step input (Fig. 6c) to include both a step on (from low to high *X*) as well as a step off (from high to low *X*):

22 $$X(T)={X}_{0}+({\eta }_{X}-{X}_{0})\left({{\Theta }}(T)-{{\Theta }}(T-\omega )\right)$$

where the Heaviside function is defined such that Θ(*T*) = 0 for *T* < 0 and Θ(*T*) = 1 for *T* > 0. Eq. (22) represents a square input pulse of width *ω* starting at *X*_0_ and reaching to *η*_*X*_. This is an on-pulse if *η*_*X*_ > *X*_0_ and an off-pulse if *η*_*X*_ < *X*_0_.

The lack of feedback in feedforward loops and the fact that the square-pulse input of Eq. (22) takes on only two values simplify the form of Eq. (20) significantly, allowing it to be solved explicitly (Supplementary Note 6). The solution is:

23 $$Z(T)={Z}_{ss}+{\mathrm{sgn}}\,({n}_{1}){\eta }_{1}{Z}_{1}^{{n}_{1}}f(T)+{\mathrm{sgn}}\,({n}_{2}){\eta }_{2}{Z}_{K}^{{n}_{2}}f(T-{{\Delta }}\gamma )$$

in which

24 $$f(T) 	=\left(1-{e}^{-T}\right){{\Theta }}(T)-\left(1-{e}^{-(T-\omega )}\right){{\Theta }}(T-\omega )\\ {Z}_{ss} 	=\frac{{\eta }_{1}}{1+{X}_{0}^{{n}_{1}}}+\frac{{\eta }_{2}}{1+{\left(K{X}_{0}\right)}^{{n}_{2}}}\\ {Z}_{\kappa }^{n} 	=\frac{1}{1+{\left(\kappa {\eta }_{X}\right)}^{| n| }}-\frac{1}{1+{\left(\kappa {X}_{0}\right)}^{| n| }}.$$

Here, *Z*_*ss*_ is the steady-state value of *Z*, ${Z}_{\kappa }^{n}$ are the magnitudes of deviation from steady state due to each arm, and *f*(*T*) specifies where the responses from the two regulation arms are active. In terms of the original *X* (as opposed to the time-shifted $\hat{X}$), the output in Eq. (24) is shifted to the right in time by *γ*_1_ relative to the original input *X*(*T*).

There are several interesting results to note in this equation. First, the cooperativities (*n*_1_, *n*_2_) and thresholds (*K*) affect the response magnitude, but not the dynamics (*f*), because the input signal is already infinitely steep.

Second, the *η* values only affect the steady-state *Z*_*ss*_ and the response logic (as ${\mathrm{sgn}}\,({n}_{i}){\eta }_{i}$, see logic discussion). Only 8 logic gates correspond to feedforward loops, in that they respond to both inputs: the four AND-type functions and four OR-type functions.

Third, *f* indicates where responses are active, varying from 0 far outside a pulse, aprroaching 1 inside for wide pulses (*ω* ≫ 0). The output then has four responses (pulse start and pulse end for each arm), in which *Z* moves away from *Z*_*ss*_ by as much as ${Z}_{\kappa }^{n}$ (pulse on) before returning (pulse off). Responses must be driven externally, unlike autoregulation.

For coherent feedforward motifs, input pulses must be sufficiently wide (*ω* ≫ 1) to fully activate the output *R*. This implies that coherent feedforward motifs act as filters against short signals, either short on signals (AND and NAND) or short off signals (OR and NOR). The delay difference Δ*γ* determines how quickly the response starts (AND and NOR logic) or returns to baseline once the pulse is over (for OR and NAND logic), so that large Δ*γ* maintains a response long after the input pulse has subsided.

For incoherent feedforward motifs, the output *R* is only fully activated if the pulse is wide (*ω* ≫ 1) and the delay difference is also wide (Δ*γ* ≫ 1), in which case a pulse of output is formed. The output pulse is formed on either the on-step (slow arm IMPLY fast arm, and slow arm NIMPLY fast arm) or the off-step (fast arm IMPLY slow arm, and fast arm NIMPLY slow arm). This means that incoherent feedforward motifs act as pulse generators. The delay difference Δ*γ* determines the minimum width of input pulse that will produce a response in *R*.

Many of the feedforward behaviors described in this section corroborate earlier results from ODE models^2,2,58,74,75,77,77,78^, but are derived here with just a single equation. In particular, it is well known that coherent feedforward loops have filter capabilities and that incoherent feedforward loops can act as pulse generators (temporal^2,58,77^ or spatial^79^). We did not demonstrate fold-change detection (FCD)^78^ because of our use of sum-like logic; an alternative logic formulation does show FCD, implying that FCD has certain mechanistic requirements (Supplementary Note 6). Furthermore, we clearly demonstrated that the feedforward loop dynamics depend entirely on the difference of delays in the two regulation arms, a fact that can only be seen indirectly with ODE models^58^.

#### Frequency response of feedforward motifs can be solved analytically and demonstrates low- and band-pass filtering capabilities

Biological regulatory networks often encode information as the change in frequency of an oscillating input^80^, which has been suggested to be more robust to noise than encoding information in absolute concentration^81–83^. That feedforward loops filter short square pulses suggests more general frequency-filtering capabilities. We therefore analyze feedforward frequency response to sinusoidal input (Fig. 6d–g). We show that the response follows the outline of a universal transfer function curve, independent of the logic or delay difference.

Instead of the step input analyzed above, here we consider a sinusoidal input

25 $$X(T)=A(1+\cos (2\pi fT))$$

which oscillates between zero and 2*A* (twice the amplitude) at a frequency *f* > 0. Taking the Fourier decomposition of each Hill-regulated term 1/[1 + *X*^*n*^(*T*)] and plugging into the governing Eq. (21) (Supplementary Note 6) provides the output *Z*(*T*) in terms of its magnitude *I*_*k*_ and phase *ϕ*_*k*_ as a function of frequency:

26 $$Z(T) 	=\frac{{\eta }_{1}{a}_{0}^{x;{n}_{1}}+{\eta }_{2}{a}_{0}^{x;{n}_{2}}}{2}+\mathop{\sum }\limits_{k=1}^{\infty }{I}_{k}\cos (2\pi kfT-{\phi }_{k})\\ {I}_{k} 	=\sqrt{\frac{{({\eta }_{1}{a}_{k}^{x;{n}_{1}})}^{2}+2{\eta }_{1}{\eta }_{2}\cos (2\pi kf{{\Delta }}\gamma ){a}_{k}^{x;{n}_{1}}{a}_{k}^{x;{n}_{2}}+{({\eta }_{2}{a}_{k}^{x;{n}_{2}})}^{2}}{1+{(2\pi kf)}^{2}}}\\ {\phi }_{k} 	={\rm{atan2}}\left(2\pi kf{\eta }_{1}{a}_{k}^{x;{n}_{1}}+{\eta }_{2}(2\pi kf\cos (2\pi kf{{\Delta }}\gamma )+\sin (2\pi kf{{\Delta }}\gamma )){a}_{k}^{x;{n}_{2}},\right.\\ 	\qquad\qquad \left.{\eta }_{1}{a}_{k}^{x;{n}_{1}}+{\eta }_{2}(\cos (2\pi kf{{\Delta }}\gamma )-2\pi kf\sin (2\pi kf{{\Delta }}\gamma )){a}_{k}^{x;{n}_{2}}\right),$$

where ${a}_{k}^{x;n}$ are Fourier coefficients of Hill-regulated terms 1/[1 + *X*^*n*^(*T*)] at integer multiples *k* of the fundamental frequency *f*:

27 $${a}_{k}^{x;n}=\frac{1}{\pi }\mathop{\int}\nolimits_{0}^{2\pi }\ \frac{\cos (kT)}{1+{A}^{n}{(1+\cos (T))}^{n}}\ {\rm{d}}T,$$

for all *f* > 0. Note that the magnitudes are symmetric to interchange of the two regulation arms (*η*_1_ ↔ *η*_2_, *n*_1_ ↔ *n*_2_), while the phases are not.

Looking at frequencies that are integer multiples of 1/Δ*γ*, we can see that both the magnitudes and phases follow a universal envelope not dependent on delays or logic (i.e., no dependence on *γ* or *η* values):

28 $$\begin{array}{lll}{I}_{k,{\rm{env}}}={I}_{0}/\sqrt{1+{(2\pi kf)}^{2}}\\ {\phi }_{k,{\rm{env}}}={\tan }^{-1}\left(2\pi kf\right)-{\phi }_{0}\end{array}$$

where (from Eq. (26), at *k* = 0 or *f* → 0) ${I}_{0}={\eta }_{1}{a}_{k}^{x;{n}_{1}}+{\eta }_{2}{a}_{k}^{x;{n}_{2}}$ and ${\phi }_{0}=\pi ({\mathrm{sgn}}\,{I}_{0}+1)/2$. The actual magnitudes and phases are then modulated from these envelopes with period 1/*k*Δ*γ*. The fundamental-frequency (*k* = 1) response (the largest magnitude, see Fig. 6d, e) are shown compared to the envelopes in Fig. 6f (known as Bode plots^76^).

At frequencies *f* that are integer multiples of 1/*k*Δ*γ*, the cosine in *I*_*k*_ equals ±1. Because ${a}_{k}^{x;-n}=-{a}_{k}^{x;n}$ (see Eq. (27), Supplementary Note 6), the output magnitude *I*_*k*_ then increases to a local maximum for coherent feedforward motifs (${\mathrm{sgn}}\,({n}_{1})={\mathrm{sgn}}\,({n}_{2})$) and decreases to a local minimum for incoherent feedforward motifs (${\mathrm{sgn}}\,({n}_{1})=-{\mathrm{sgn}}\,({n}_{2})$). The opposite holds for frequencies that are half-integer multiples of 1/*k*Δ*γ*. For the special case of perfectly balanced incoherent feedforward motifs (*η*_1_ = *η*_2_, *n*_1_ = −*n*_2_, *K* = 1), the magnitudes *I*_*k*_ decrease to zero for frequencies that are half-integer multiple of 1/Δ*γ*; otherwise, the maxima (for coherent) and minima (for incoherent) equal *I*_*k*_(*f* = 0).

Incredibly, the envelopes (relative to *f* = 0) have zero parameters, making them universal to all feedforward loops. The frequency at which the magnitude decreases to one-half its value at *f* = 0 occurs at $f=\sqrt{3}/2\pi k$, with a phase shift of −*π*/3.

Despite the non-dependence on Δ*γ*, the absolute maximum magnitude obtained for incoherent feedforward motifs does depend on Δ*γ*, as does the frequency at which this maximum response occurs. The dependence on delay difference is particularly strong near Δ*γ* = 1 (Fig. 6g). This is because for Δ*γ* ≫ 1, the envelope decreases by half when *f* is many multiples of Δ*γ*, and thus past many local maxima. The local maximum then occurs at 0 < *f* ≪ 1 with a maximum above *I*_*k*_(*f* = 0). For Δ*γ* ≪ 1, on the other hand, the envelope decreases by half before a single sinusoid is complete, so the maximum is at *f* → 0.

At Δ*γ* ≈ 1, the first sinusoid reaches its maximum while the envelope is descending most steeply, and the maximum occurs at *f* ≈ 1 with a value highly dependent on Δ*γ*. To activate *R*, *X* must go above 1 for at least part of the output oscillation, meaning that *I*_1_ must go above 1 − 〈*I*〉 = 1 − *I*_0_. Looking at Fig. 6g, we see that for large Δ*γ* ≫ 1, this occurs for small frequencies, and the feedforward loop acts as a low-pass filter. However, there is a window of Δ*γ* ≈ 1 in which 1 − *I*_0_ is large, at modest frequencies (0.1 < *f* < 10), corresponding to the hump in magnitude, e.g., around *f* = 0.5 for Δ*γ* = 1 in Fig. 6f. The incoherent feedforward motif thus acts as a bandpass filter for Δ*γ* ≈ 1, with ineffective regulation outside of modest frequencies.

For coherent feedforward motifs, the frequency at which the first minimum in output response occurs follows the same logic, but the maximum response is always found at *f* → 0. Since the maximum response is at low frequencies, coherent feedforward motifs act as low-pass filters, as has been shown to be a basic expected behavior of chemical networks^84^.

### Motif V: multi-component feedback

**Fig. 7:**
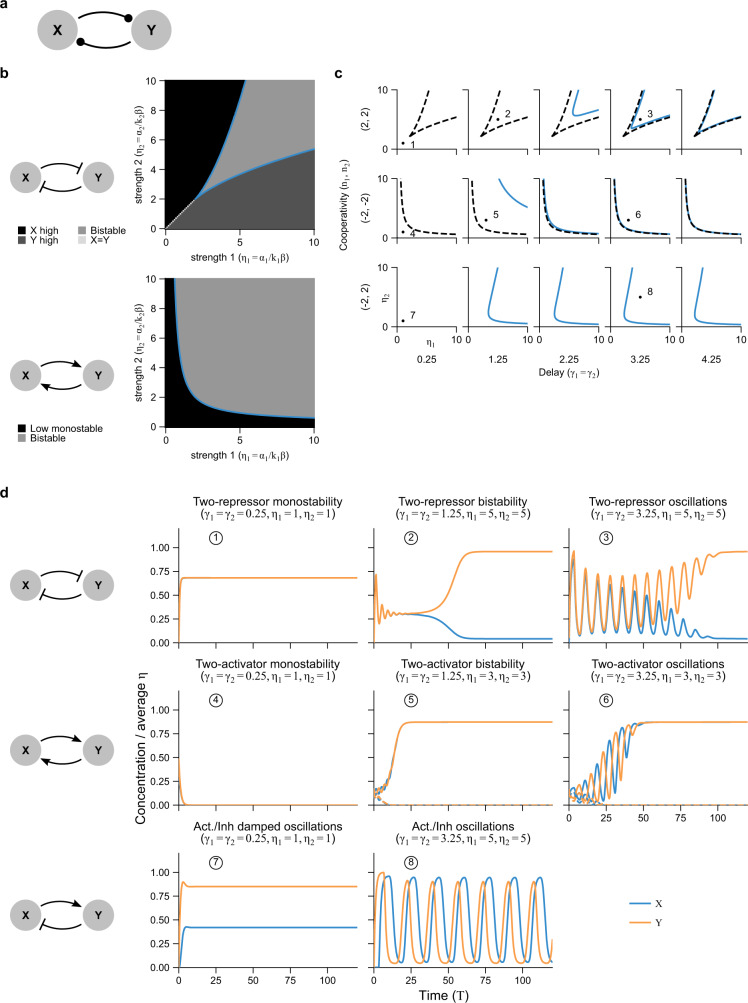
Two-component autoregulatory loops reproduce behaviors of autoregulation, but have additional behaviors describing the relative dynamics of the components. **a** A two-component loop network motif, which is similar to autoregulation but with two explicitly modeled genes instead of one. **b** Parameter space of the two regulatory strength parameters showing phase diagram for a loop composed of two activators (cross-activating) or two repressors (cross-inhibitory). Shading shows results of simulations (with an interval of 0.1 for both *γ* and *η* axes); blue curve is the analytically derived phase (bifurcation) boundary from Eq. (32). **c** Parameter space varying both strength parameters. Because the Hopf bifurcations depend only on the total delay and transient oscillations most prominent for equal delays, we show only the cases *γ*_1_ = *γ*_2_. Blue curves show analytically derived Hopf bifurcations. Black dashed curves are the bifurcation boundaries from (**b**). Except for the activator/repressor case, all these curves lie above the bistability boundary given by the black curve in (**b**), meaning oscillations are always transient. **d** Representative simulations for specific initial conditions showing all possible qualitative behaviors for a two-component loop with two activators, two repressors, or one activator and one repressor. For two-activator bistability and oscillations, a second set of initial conditions is shown in dashed lines to demonstrate the bistability. Hill coefficients equal 2 for repressors, −2 for activators.

The common two-component feedback motif^58^ is the equivalent of a cascade with feedback (Fig. 7a). We can write down the governing equations for such a system, using the same normalizations as before (see Supplementary Note 7), assuming for simplicity zero leakage and that *x* and *y* have the same degradation rates *β*. This is equivalent to the 3-step cascade Eq. (8) with equal degradation rates and setting *Z* = *X* to form the loop.

29 $$\begin{array}{lll}\dot{X}(T)=\frac{{\eta }_{1}}{1+{Y}^{{n}_{1}}(T-{\gamma }_{1})}-X(T)\\ \dot{Y}(T)=\frac{{\eta }_{2}}{1+{X}^{{n}_{2}}(T-{\gamma }_{2})}-Y(T).\end{array},$$

The fixed points are given by ${\eta }_{1}=X(1+{Y}^{{n}_{1}})$, ${\eta }_{2}=Y(1+{X}^{{n}_{2}})$ or (for two activators) *X* = *Y* = 0. Linearizing around these fixed points and assuming ansatz solutions of the form $\delta X(T)=A\exp ({\lambda }_{1}T),\delta Y(T)=B\exp ({\lambda }_{2}T)$, we find the set of characteristic equations

30 $$\begin{array}{lll}A(\lambda +1)+{\eta }_{1}{M}_{1}B{e}^{-\lambda {\gamma }_{1}}=0\,\\ B(\lambda +1)+{\eta }_{2}{M}_{2}A{e}^{-\lambda {\gamma }_{2}}=0.\end{array}$$

where *λ*_1_ = *λ*_2_ ≡ *λ* must hold for the ansatz to be true for all time. This can be rewritten in matrix form, with a matrix *J* times the vector (*A*, *B*)^⊤^.

Diagonalizing *J* results in two characteristic equations (Supplementary Note 7)

31 $${{{\Lambda }}}_{\pm }\equiv \lambda \pm \sqrt{{\eta }_{1}{M}_{1}}\sqrt{{\eta }_{2}{M}_{2}}{e}^{-\lambda ({\gamma }_{1}+{\gamma }_{2})/2}+1=0$$

for two corresponding eigenmodes ${v}_{+}={(1,R{e}^{i\phi })}^{\top }$ and ${v}_{-}={(-1,R{e}^{i\phi })}^{\top }$, respectively, where *B*/*A* = *Re*^*iϕ*^ is the ratio of *δX* to *δY* components in magnitude-phase notation (Supplementary Note 7). Overall, the eigenvectors indicate a phase difference between *X* and *Y* of *ϕ* for *v*_+_ and *ϕ* + *π* for *v*_−_. When the Λ_+_ version of Eq. (31) is satisfied for $\mathrm{Re}\,\lambda =0$, *v*_+_ bifurcates and when the Λ_−_ version is satisfied for $\mathrm{Re}\,\lambda =0$, *v*_−_ bifurcates.

#### Saddle-node bifurcations determine bistability modes

For saddle-node bifurcations, at which bistability begin, we set *λ* = 0. This can only be satisfied by the Λ_+_ version of Eq. (31) if *n*_1_ < 0, *n*_2_ < 0 (two activators), and by the Λ_−_ version if *n*_1_ > 0, *n*_2_ > 0 (two repressors). A feedback loop of an odd number of repressors cannot have a saddle-node bifurcation. As for linear cascades (Fig. 3), the overall autoregulation is repressive only if it contains an odd number of repressors. The bistability boundary for both cases is then given by (Supplementary Note 7):

32 $${\eta }_{1} 	={n}_{1}{n}_{2}\frac{{X}^{{n}_{2}+1}}{({n}_{1}{n}_{2}-1){X}^{{n}_{2}}-1}\\ {\eta }_{2} 	={n}_{1}{n}_{2}\frac{{Y}^{{n}_{1}+1}}{({n}_{1}{n}_{2}-1){Y}^{{n}_{1}}-1}\\ f(X,Y) 	\equiv \frac{{n}_{1}{n}_{2}{X}^{{n}_{2}}{Y}^{{n}_{1}}}{\left(1+{X}^{{n}_{2}}\right)\left(1+{Y}^{{n}_{1}}\right)}=+1$$

where *f* = 1 provides an implicit monotonic mapping between *X* and *Y* that restricts *η*_1_(*X*) with respect to *η*_2_(*Y*).

The phase difference *ϕ* = 0, implying that two-activator loops show bistability with *X* and *Y* either both high or both low, because the components of *v*_+_ are of like sign, whereas two-repressor loops (*v*_−_) show bistability with *X* high and *Y* low or vice versa (Fig. 7b, d).

#### Hopf bifurcations determine transient oscillations in modes restricted by bistability

For Hopf bifurcations, at which oscillations begin, we set *λ* = *iω*. The boundaries are given for both eigenmodes by (Supplementary Note 7)

33 $${\gamma }_{1} =	\frac{1}{\omega }\left[-{\tan }^{-1}\omega +\phi +\frac{\pi }{2}(1-{\mathrm{sgn}}\,{n}_{1})+\pi k\right],\ k=0,1,\ldots \\ {\gamma }_{2} =	\frac{1}{\omega }\left[-{\tan }^{-1}\omega -\phi +\frac{\pi }{2}(1-{\mathrm{sgn}}\,{n}_{2})+\pi k\right],\ k=0,1,\ldots \\ {\eta }_{1} =	\frac{|{n}_{1}{n}_{2}| }{1+{\omega }^{2}}\cdot \frac{{X}^{{n}_{2}+1}}{\left(\frac{| {n}_{1}{n}_{2}| }{1+{\omega }^{2}}-1\right){X}^{{n}_{2}}-1}\\ {\eta }_{2} =	\frac{| {n}_{1}{n}_{2}| }{1+{\omega }^{2}}\cdot \frac{{Y}^{{n}_{1}+1}}{\left(\frac{| {n}_{1}{n}_{2}| }{1+{\omega }^{2}}-1\right){Y}^{{n}_{1}}-1}\\ f(X,Y) \equiv 	\frac{| {n}_{1}{n}_{2}| }{1+{\omega }^{2}}\cdot \frac{{X}^{{n}_{2}}{Y}^{{n}_{1}}}{\left(1+{X}^{{n}_{2}}\right)\left(1+{Y}^{{n}_{1}}\right)}=1$$

where again *f* = 1 provides an implicit monotonic mapping *η*_1_(*X*) and *η*_2_(*Y*).

These curves lie above the bistability boundary, so oscillations in *v*_+_ are not observed for two activators nor in *v*_−_ for two repressors. The alternate eigenmode does show oscillations in each case (*k* = 0, *v*_−_ for activators and *k* = 1, *v*_+_ for repressors), but these oscillations are transient due to existence of bistability-induced, distant stable fixed points. For one repressor and one activator, sustained oscillations begin at *k* = 0 for *v*_−_, with *ϕ* = −*π*/2 if *n*_1_ > 0, *n*_2_ < 0 (*Y* leads *X*) and *ϕ* = *π*/2 if *n*_1_ < 0, *n*_2_ > 0 (*Y* lags *X*).

Based on Eq. (33), we can see that the primary Hopf bifurcations are equivalent for differing values of the delays as long as the average delay is equal, because the oscillation frequency *ω* of the eigenmodes only depends (implicitly) on the average delay:

34 $$\langle \gamma \rangle \equiv \frac{1}{2}\left({\gamma }_{1}+{\gamma }_{2}\right)=\frac{1}{\omega }\left(-{\tan }^{-1}\omega +\frac{\pi }{4}(2-{\mathrm{sgn}}\,{n}_{1}-{\mathrm{sgn}}\,{n}_{2})+\pi k\right).$$

In particular, oscillations are possible for activator-repressor loops even when one delay is zero, as long as the sum of delays is greater than zero. On the other hand, the phase difference between the oscillations depends only on the difference in delays given the value *ω*:

35 $$\phi =\frac{\omega }{2}({\gamma }_{1}-{\gamma }_{2})+\frac{\pi }{4}\left({\mathrm{sgn}}\,{n}_{1}-{\mathrm{sgn}}\,{n}_{2}\right)$$

Together with the additional phase difference of *π* for *v*_−_, this implies that for equal delays, there are synchronous oscillations for two-repressor loops, anti-synchronous oscillations for two-activator loops, and *π*/2-shifted oscillations for activator-repressor loops. This phase relation also holds off the bifurcation boundary, where *λ* = *μ* + *iω*, as *ϕ* does not depend on *μ* (Supplementary Note 7). Note also that the overall phase in the expressions for *γ* are 0 for two repressors, *π* for two activators, and *π*/2 for activator-repressor loops.

Finally, one can also conclude from Eq. (30) that (Supplementary Note 7)

36 $$\lambda =\frac{1}{{\gamma }_{2}-{\gamma }_{1}}\mathrm{ln}\,\left(\frac{{\eta }_{1}{M}_{1}{B}^{2}}{{\eta }_{2}{M}_{2}{A}^{2}}\right),$$

which holds for both the real and imaginary parts of *λ*. This implies that the oscillations grow most quickly and have the highest frequency when the delays are nearly equal (*γ*_1_ = *γ*_2_). This consequently makes the transients most noticeable for equal delays. All these results can be seen in the simulated data (Fig. 7b,d).

### Motif VI: double feedback

**Fig. 8:**
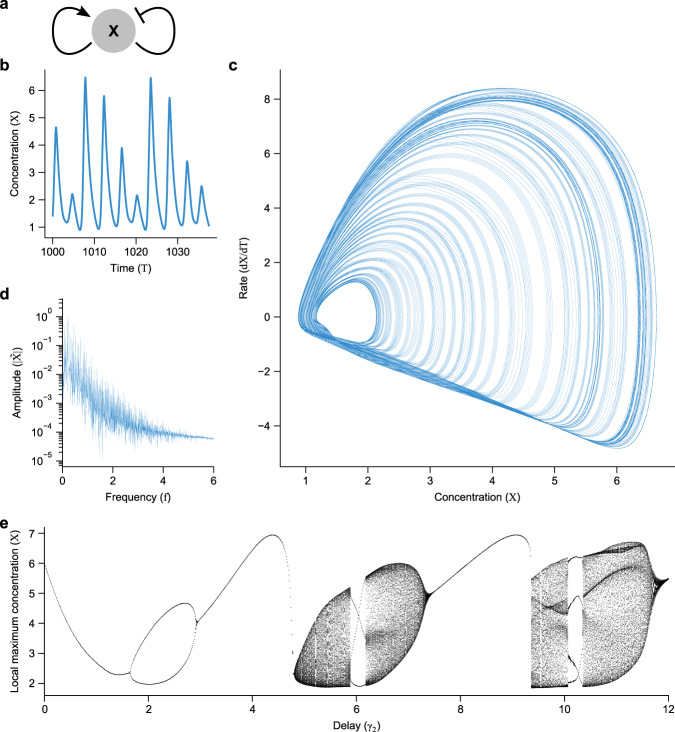
Positive/negative dual feedback can induce chaotic behavior when the difference in delay times is significant. **a** The double feedback motif, in which two regulation arms feed back directly, each with its own explicit delay. Here we show one arm activating and one repressing; for double repressive feedback, see Supplementary Fig. 5. **b** Time trace of chaotic dynamics after initial transients. **c** Trace of dynamics in phase space, with the derivative on the vertical axis. While a simple oscillator would trace a loop (possibly with multiple sub-loops if the waveform is complicated), the chaotic dynamics appear to trace out a fractal attractor. Consistent with chaos, a reconstructed phase space with coordinates (*X*(*T*), *X*(*T* − 10), *X*(*T* − 20)) traces out a fractal attractor, with box dimension 1.81 with 95% confidence interval (1.76, 1.87) and a positive dominant Lyapunov exponent (0.0040 ± 0.00055 bits), see “Methods” for details. **d** Fourier transform of chaotic dynamics show many peaks, indicating that there is no simple set of frequencies underlying the dynamics. **e** Bifurcation diagram for double positive/negative feedback, with local maxima plotted. Simple oscillations intersperse regimes with complex dynamics, where local maxima with a range of values are found. *η*_1_ = 15, *η*_2_ = 1, *n*_1_ = 11, *n*_2_ = −3, and *γ*_1_ = 1. For (**b**–**d**), *γ*_2_ = 11.

Multiple feedback (Fig. 8) has been described to lead to interesting dynamics, such as excitablity^85^, quasiperiodicity^71^, and chaos^71^. A full analysis of this motif is beyond the scope of this work, for reasons described below.

We focus on chaos, a dynamical behavior characterized by irregular, unpredictable oscillations^45^. It has been suggested that biological systems avoid chaos^86,87^, but also that chaos can describe pathological dynamics such as irregular breathing^26^ and epilepsy^27^. There may also be cases where chaos is important to biological function^88–91^. Mathematical models with^71,92,93^ and without^86,94^ delays can be chaotic.

#### Chaotic behaviors are prevented in monotonic regulation with linear or cyclic network topology

Due to cyclic network topology (no more than one loop) and monotonic regulation, none of the motifs so far can yield chaotic dynamics^46^. Either non-monotonic feedback (as in the Mackey-Glass equation^26^) or non-cyclic topology^71^ is required for chaos. Double feedback (Fig. 8a) is a minimal motif fulfilling both requirements, although the non-monotonicity is sufficient (Supplementary Note 8).

The governing equation for such a double feedback motif is thus given by setting the output and both inputs of the logic equation (Eq. (19)) to *X* (and letting *K* = 1 for simplicity):

37 $$\dot{X}(T)=\frac{{\eta }_{1}}{1+{X}^{{n}_{1}}(T-{\gamma }_{1})}+\frac{{\eta }_{2}}{1+{X}^{{n}_{2}}(T-{\gamma }_{2})}-X(T).$$

#### Chaotic behavior is possible for non-monotonic feedback with multiple feedback and disparate delays

Simulation of Eq. (37) demonstrates chaos for some parameters of positive/negative mixed feedback, as evidenced by sustained oscillations with variable maxima (Fig. 8b), occupying an apparently fractal region in phase space (Fig. 8c), and a large number of peaks in frequency space (Fig. 8d). To confirm that the dynamics are in fact chaotic, we calculated the dominant Lyapunov exponent using the Wolf method^95^ (see “Methods”), finding a positive (i.e., chaotic) value of 0.0040 ± 0.00055 bits (mean ± standard deviation). We also calculated the box dimension of a reconstructed phase space with coordinates (*X*(*T*), *X*(*T* − 10), *X*(*T* − 20)) used by the Wolf method, yielding fractional dimension ~1.81, with 95% confidence interval (1.76, 1.87). These results indicate chaos (see Supplementary Fig. 4).

Varying *γ*_2_ relative to *γ*_1_ = 1 (Fig. 8e) shows regions of presumably chaotic dynamics interspersed with simple oscillation, as found in many chaotic systems^45^. In particular, we only find chaos if the delays in the two arms differ substantially. Both delays appear to be important, not only their ratio or difference, since no oscillations exist when both delays are less than one. A full exploration of the two-delay parameter space is left to future work.

In constrast, we found quasiperiodic dynamics for dual negative feedback (Supplementary Fig. 5). Qualitatively, the time dynamics appear less pulsatile (Supplementary Fig. 5b) than dual positive/negative feedback, in line with reports that dual positive/negative feedback demonstrates excitability^85^. The attractor also has a somewhat different profile (Supplementary Fig. 5c) that in reconstructed space (*X*(*T*), *X*(*T* − 10), *X*(*T* − 20)) traces out a torus with box dimension ~2 and near-zero Lyapunov exponent (see Supplementary Fig. 4), indicating quasiperiodic rather than chaotic dynamics. The Fourier spectrum also exhibits a less dense set of peaks (Supplementary Fig. 5d).

Many parameter choices can prevent chaos in dual feedback. Double autoactivation (*n*_1_ < 0, *n*_2_ < 0) prevents chaos via a stable fixed point (*X* = 0)^45^. Identical regulation strength and cooperativity (*η*_1_ = *η*_2_, *n*_1_ = *n*_2_) behaves like autoregulation close to fixed points, with *γ* = *γ*_1_ + *γ*_2_, as do identical delays (*γ*_1_ = *γ*_2_), with *ηM* = *η*_1_*M*_1_ + *η*_2_*M*_2_ (Supplementary Note 8). Thus, for chaotic solutions to occur via double feedback, there must be at least one negative feedback arm, the delays must differ, and either the cooperativities or strengths must differ. If biological systems evolved to avoid chaos^86,87^, it may be that these conditions are selected against, even if the double-feedback motif is not.

The complete parameter space for double feedback is likely to be highly complex, with boundaries possibly formed via an infinite number of bifurcation curves^48^. In particular, while we demonstrated chaotic and quasiperiodic dynamics for specific parameter values, a more in-depth exploration is warranted to fully understand the dynamics for all parameters. For further exploration of double negative feedback and more complex delayed network motifs that exhibit chaotic dynamics, see Suzuki et al.^71^.

## Discussion

In this work, we systematically studied the most common network motifs (Table 2) by use of DDEs. Compared to the more widely used mechanistic ODE descriptions, we demonstrated numerous instances where inclusion of explicit delays simplifies complex systems into smaller motifs while continuing to capture their key dynamics that are otherwise lost in simplifying complex systems as small motifs. To further modeling simplicity, we also identified unifying descriptions for activators and repressors (Eq. (7)) and for boolean Hill function logic (Fig. 5) that are useful for both DDE and ODE models.

**Table 2:** Summary of differences between ODE and DDE network regulation models and key findings, and where each DDE cartoon provides a simplified, unified model for several ODE cartoons.

Motif	ODE Cartoons	DDE Cartoons	Equations	Key Findings
0: Direct regulation			Eq. (5)	Activators and inhibitors combined via negative Hill coefficient. Delay provides explicit timescale. Figure 2.
I: Cascade			Eq. (9)	ODE cascades can be reduced to simple DDE regulation. Delays sum. Figure 3.
II: Autoregulation			Eq. (11)	Full parameter space derived. Delays matter only for negative autoregulation. Figure 4, Supplementary Fig. 3, Supplementary Fig. 1, Supplementary Fig. 2.
III: Logic			Eq. (19)	Sum of Hill terms yields all monotonic logic gates in one parameter space. Delays not important for steady states, only strengths. Figure 5, Supplementary Table 2, Supplementary Table 1.
IV: Feedforward loop			Eqs (20), (21)	Capable of pulsing, signal filtering by input pulse width or frequency. All signal processing behavior is due to logic and difference in delays between arms. Figure 6.
V: Feedback loop			Eq. (29)	Delay sum governs presence of oscillations, which are transient for two repressors (synchronous) and two activators (anti-synchronous). Delay difference governs phase. Full parameter space derived. Figure 7.
VI: Double feedback			Eq. (37)	Chaos and quasiperiodicity possible for two-delay feedback and not for simpler motifs^46,71^. Figure 8, Supplementary Fig. 5. Future work.
Complex networks			Eqs S84, S85, S87	Matrix analysis available (Supplementary note 9, Glass, et al.^23^). Complex dynamics and spatial behaviors possible. Future work. Supplementary Fig. 6.

There are multiple findings to highlight. First, multistep cascades with and without feedback reduce effectively to direct, delayed regulation, providing an intuitive interpretation of biological delays as resulting from multi-step processes, and establishing analytical relationships for converting between parameters of the corresponding ODE and DDE models. Second, delays determine many key motif properties, such as the oscillation period in negative autoregulation, and pulse width and frequency cutoff in feedforward motifs. Interplay of multiple delays plays a similar role in multi-component feedback for determining absolute and relative oscillation periods, and in multiple feedback for determining chaotic dynamics. Third, we showed quantitatively not only when delays are crucial to behavior, as in oscillations, but also when they may be safely ignored. Delays do not affect steady states or logic of independent inputs, only a difference of delays between regulation arms is important in determining feedforward behavior, and only the sum of delays determines the onset of transient oscillations in feedback loops.

Using DDE models to contract a network into a much smaller network with equivalent topology (replacing all cascades with delayed, direct regulation) may aid in discovery of large-scale motifs that have important functions or that might otherwise be perceived as statistically insignificant. The motifs in this paper were originally discovered by scanning biological networks for subnetworks of *N* nodes that occur more frequently than expected by chance^6,11^ (this in fact being the original definition of network motifs). However, this discovery method becomes increasingly difficult for *N* ≳ 5 due to the combinatorial scaling of the number of possible motifs^96^. Performing a similar search on contracted networks would be equivalent to searching the original network for larger motifs, and places a stronger emphasis on topology than on number of components involved (e.g., *X* → *Z* being equivalent to *X* → *Y* → *Z*). While this can be done without DDEs, the introduction of delays allows one to perform the contraction while maintaining key information about the original dynamics of retained components.

While our work has focused on the most basic network motif topologies (Table 2), the same techniques can be applied to other more complex networks. Expanding on the eigenmode analysis we performed for multi-component feedback loops, one can describe the linear behavior of an arbitrary network near its fixed points by a matrix form of the characteristic equation $J{\mathbf{a}}=0$ with ${J}_{ij}=(\lambda +1){\delta }_{ij}+{\eta }_{j}{M}_{j}{e}^{-{\gamma }_{ij}\lambda }$ (Supplementary note 9, Supplementary Fig. 6). This approach allows simple models to provide detailed predictions of complex biological phenomena such as pattern formation^23^.

To clarify the basic role of delays in network motifs, we ignored several phenomena which we suggest that future work explore. These include: (1) Noise, which is intrinsic to many biological systems^97^ and known to affect dynamics of delay equations^15,92,98,99^. (2) Time-varying and stochastic delays^42,47,98,100^. (3) Non-constant initial conditions (“histories”) for *T* < 0; for example, autorepression shows in-phase and anti-phase locking with constant histories, while sine-wave histories with randomized phase do not (Supplementary Fig. 7). (4) Non-Hill regulation and complex degradation functions^26,78^, such as zeroth-order^101^, nonlinear^102^, or delayed removal, as well as diffusion or spatial effects^103^. (5) Feedback with multiple delays, which we only briefly analyzed in the double feedback motif, and likely have very complicated parameter spaces^48,49^.

Overall, we believe that our work may help resolve fundamental biological and engineering questions regarding a variety of phenomena, including transcription factor networks^6,17,77^, cell cycles^87^, other biological clocks^14,18,25^, and pattern formation^23,44^. Multiple feedback analysis may determine whether chaos affects the cell cycle^87^, or whether biology avoids possibly chaotic motifs. Delays in multicellular signaling^23,32^ may distinguish among models of pattern formation^16,24,32,103,104^. Finally, exploring “contracted” networks with delays may uncover entirely new functional network motifs that are larger and more complex than currently known^96^.

## Methods

### Analytics and numerical simulation

Analytics were in general performed by hand, and checked for validity using Mathematica. Numerical simulations were run in Matlab using the dde23 delay differential equation solver for DDEs and ode45 for ODEs. Simulating activators as repressors with *n* < 0 technically fails when *x* is identically zero (Eq. (5)), since that would imply division by zero, but the limit as *x* goes to zero causes the regulation term to be zero, which is the same result as assumed by our notation. An initial value of exactly zero for *x* can thus lead to a divide-by-zero error in simulations, and so initial conditions of exactly zero were not used, as that case is an uninteresting fixed point for activators in any case. Note also that the consitutive case for Eq. (5) is degenerate, in that *n* = 0, *α* ≠ 0 is equivalent to *n* ≠ 0, *α* = 0 with *α*_0_ → *α*_0_ + *α*/2.

### Phase plot simulations and analysis

For autoregulation phase plots, simulations were run with 100 constant-history initial conditions spread logarithmically between 10^−4^ and 2*η* and run from *T* = 0 to *T* = 100(*γ* + 1). Solutions were considered stable if for all 100 simulations the maximum absolute value of the discrete derivative in the last three-quarters of the simulation time was less than 0.1. Stable solutions were sub-categorized as bistable if a histogram of final values over all 100 solutions had more than 1 peak. Solutions were considered oscillatory if the average Fourier transform of the last three-quarters of the simulation time for all 100 solutions had more than zero peaks with amplitude (square root of power) greater than 100. Solutions were considered spiral if this oscillation condition held for the first one-quarter of the simulation time only. For two-component loops, initial conditions were used that ranged between 0 and $\max ({\eta }_{1},{\eta }_{2})$, for equal *X* and *Y* and for apposing *X* and *Y*. Bistability was determined as for autoregulation, and a cutoff of 0.05 was used to determine “low” values. All simulation histories were constant except where indicated in Supplementary Fig. 7. Specific parameter values and simulation details are given in the figures and/or made explicit in the MATLAB code in Supplementary Data 1.

### Lyapunov exponents and box dimensions

For calculating dominant Lyapunov exponents, we used the Wolf method^95^ with recommended parameters from the MATLAB script “Wolf Lyapunov exponent estimation from a time series” (version 1.2.0.1) provided by the authors on the Mathworks FileExchange (https://www.mathworks.com/matlabcentral/fileexchange/48084-wolf-lyapunov-exponent-estimation-from-a-time-series). In particular, we used an embedding dimension of 3 and a phase space reconstruction delay of 10 for a dataset with ~350 orbits of ~50–60 data points per orbit. The last 50 iterations of the algorithm were used to generate a mean and standard deviation of the estimated dominant Lyapunov exponent. A full set of parameters can be found in the included code. For calculating attractor dimensions, we used the box-counting method, whose code is also provided in Supplementary Data 1, on the reconstructed phase space (*X*(*T*), *X*(*T* − 10), *X*(*T* − 20)) used for calculating Lyapunov exponents. A linear regression was performed using MATLAB’s fit function on half the linear domain to generate a mean and confidence interval (using MATLAB’s confint) for the slope (i.e., box dimension) between numbers of boxes covering the attractor versus length of each box.

### Reporting summary

Further information on research design is available in the Nature Research Reporting Summary linked to this article.

## Supplementary information

Supplementary Information

Reporting Summary

## Data Availability

All raw data is included in the supplementary material (Supplementary Data 1). The authors can confirm that all relevant data are included in the paper and/or its supplementary information files. Source data are provided with this paper.

## Source data

Source Data

## Code availability

All code used to generate data for this work is included in the supplementary material (Supplementary Data 1). The authors can confirm that all relevant code is included in the paper and/or its supplementary information files.
