# Supplementary material for: Nonlinear delay differential equations and their application to modeling biological network motifs
Source: Nat Commun. 2021 Mar 19;12:1788. doi: 10.1038/s41467-021-21700-8 (PMC7979834; doi:10.1038/s41467-021-21700-8)
Supplement: Supplementary file 2 — Reporting Summary [file 41467_2021_21700_MOESM2_ESM.pdf]

nature research

Corresponding author(s): Ingmar Riedel-Kruse

Last updated by author(s): Jan 27, 2021

Reporting Summary

Nature Research wishes to improve the reproducibility of the work that we publish. This form provides structure for consistency and transparency in reporting. For further information on Nature Research policies, see our Editorial Policies and the Editorial Policy Checklist.

Statistics

For all statistical analyses, confirm that the following items are present in the figure legend, table legend, main text, or Methods section.

n/a	Confirmed	
☐	☒	The exact sample size (n) for each experimental group/condition, given as a discrete number and unit of measurement
☐	☒	A statement on whether measurements were taken from distinct samples or whether the same sample was measured repeatedly
☒	☐	The statistical test(s) used AND whether they are one- or two-sided<br>*Only common tests should be described solely by name; describe more complex techniques in the Methods section.*
☒	☐	A description of all covariates tested
☒	☐	A description of any assumptions or corrections, such as tests of normality and adjustment for multiple comparisons
☐	☒	A full description of the statistical parameters including central tendency (e.g. means) or other basic estimates (e.g. regression coefficient) AND variation (e.g. standard deviation) or associated estimates of uncertainty (e.g. confidence intervals)
☒	☐	For null hypothesis testing, the test statistic (e.g. F, t, r) with confidence intervals, effect sizes, degrees of freedom and P value noted<br>*Give P values as exact values whenever suitable.*
☒	☐	For Bayesian analysis, information on the choice of priors and Markov chain Monte Carlo settings
☒	☐	For hierarchical and complex designs, identification of the appropriate level for tests and full reporting of outcomes
☒	☐	Estimates of effect sizes (e.g. Cohen's d, Pearson's r), indicating how they were calculated

Our web collection on statistics for biologists contains articles on many of the points above.

Software and code

Policy information about availability of computer code

Data collection	All simulations were run using MATLAB R2018b as described in the Methods using custom scripts provided in Supplementary Data 1, based around ode45 and dde23. All code used in this study and data generated by the code is provided in Supplementary Data 1, which also includes a detailed README file on how to use the code and/or data to reproduce the figures. This includes the code for the Wolf algorithm, which is provided without modification from the software "Wolf Lyapunov exponent estimation from a time series" (version 1.2.0.1) provided by Alan Wolf on the MathWorks File Exchange (https://www.mathworks.com/matlabcentral/fileexchange/48084-wolf-lyapunov-exponent-estimation-from-a-time-series), as permitted by its license.
Data analysis	Lyapunov exponents for Supplemental Figure 4 were calculated using the software "Wolf Lyapunov exponent estimation from a time series" (version 1.2.0.1) provided by Alan Wolf on the MathWorks File Exchange (https://www.mathworks.com/matlabcentral/fileexchange/48084-wolf-lyapunov-exponent-estimation-from-a-time-series), which has also been included in Supplemental Data 1 along with its license (as permitted within said license). All other analysis and plotting was done using custom MATLAB and python scripts, which are provided in Supplementary Data 1. The above link is also provided within the Methods, as is the name and version of the software.

For manuscripts utilizing custom algorithms or software that are central to the research but not yet described in published literature, software must be made available to editors and reviewers. We strongly encourage code deposition in a community repository (e.g. GitHub). See the Nature Research guidelines for submitting code & software for further information.

Data

Policy information about availability of data

All manuscripts must include a data availability statement. This statement should provide the following information, where applicable:

- Accession codes, unique identifiers, or web links for publicly available datasets
- A list of figures that have associated raw data
- A description of any restrictions on data availability

All raw data is included in the supplementary material (Supplementary Data 1). The authors confirm that all relevant data are included in the paper and/or its supplementary information files.

Field-specific reporting

Please select the one below that is the best fit for your research. If you are not sure, read the appropriate sections before making your selection.

- [x] Life sciences
- [ ] Behavioural & social sciences
- [ ] Ecological, evolutionary & environmental sciences

For a reference copy of the document with all sections, see nature.com/documents/nr-reporting-summary-flat.pdf

Life sciences study design

All studies must disclose on these points even when the disclosure is negative.

Sample size	We had no physical samples. For Lyapunov exponent calculations, we measured the last 50 iterations of the Wolf algorithm, as described in the methods. This was sufficient by inspection to cover, for every condition tested, the entire range of fluctuations in exponent estimate after the algorithm equilibrated.
Data exclusions	We did not exclude any data points.
Replication	We did not perform any physical experiments. For Lyapunov exponents and box dimensions, we did not systematically check for replicability in other conditions; however, we did calculate both metrics for control conditions (Mackey-Glass equations, autoregulation) which match theoretical expectations and literature values as described in the text.
Randomization	There were no such samples in our study.
Blinding	There was no blinding in this study, which was not relevant to this work (e.g., no group allocation).

Reporting for specific materials, systems and methods

We require information from authors about some types of materials, experimental systems and methods used in many studies. Here, indicate whether each material, system or method listed is relevant to your study. If you are not sure if a list item applies to your research, read the appropriate section before selecting a response.

Materials & experimental systems

n/a	Involved in the study	
[x]	[ ]	Antibodies
[x]	[ ]	Eukaryotic cell lines
[x]	[ ]	Palaeontology and archaeology
[x]	[ ]	Animals and other organisms
[x]	[ ]	Human research participants
[x]	[ ]	Clinical data
[x]	[ ]	Dual use research of concern

Methods

n/a	Involved in the study	
[x]	[ ]	ChIP-seq
[x]	[ ]	Flow cytometry
[x]	[ ]	MRI-based neuroimaging
