# Supplementary material for: Nonlinear delay differential equations and their application to modeling biological network motifs
Source: Nat Commun. 2021 Mar 19;12:1788. doi: 10.1038/s41467-021-21700-8 (PMC7979834; doi:10.1038/s41467-021-21700-8)
Supplement: Supplementary file 3 — Source Data [file 41467_2021_21700_MOESM3_ESM.zip › DataS1/matlab/wolflyapunovexponent/lyapunews.pdf]

# Estimating the dominant Lyapunov exponent from a Time Series

## Documentation for the efficient version

## of the algorithm of Wolf et al.

Alan Wolf
Professor of Physics
The Cooper Union
41 Cooper Square
NY, NY 10003

awolf.physics@gmail.com

**Index**

3 ...................... History of the Algorithm
4 ...................... Chaos and Lyapunov exponents
5 ...................... Time delay reconstruction
6 ...................... Introduction to FET & BASGEN
8 ...................... Quick Start
9 ...................... More on BASGEN
11 ...................... More on FET
13 ...................... Running the sample files
15 ...................... Interpreting graphical and text output
17 ...................... The ideal time series
19 ...................... Interpreting the exponent estimate
20 ...................... Comments, Bug Fixes, Enhancements
24...................... Bibliography

Acknowledgements: This algorithm was created by the author as part of his doctoral research in the physics department at the University of Texas at Austin in the early 1980's. Mark Haye invented the database algorithm used in BASGEN. Thanks to Profs. Harry Swinney and Jack Swift and to graduate student John Vastano for their assistance in developing this algorithm. Thanks to Taehyeun Park, The Cooper Union, EE'15 for converting the original Fortran code to Matlab.

## History of the algorithm

Thirty years after it was created, our algorithm for estimating the dominant Lyapunov exponent from a time series has been implemented for Matlab and has been posted on Mathworks Matlab Central File Exchange:
http://www.mathworks.com/matlabcentral/fileexchange/
More specifically, at: http://www.mathworks.com/matlabcentral/fileexchange/48084-lyapunov-exponent-estimation-from-a-time-series

Since the algorithm was first published in Physica D in 1985,1 implementations in Fortran, C, and other languages have been distributed by myself and by other individuals. See, for example:
Athanasios Margaris and Manos Roumeliotis, "A Windows Interface for the Wolf Algorithm" at https://www.academia.edu/1117844/A_Windows_Interface_for_the_Wolf_Algorithm which presents a Windows Visual C++ interface to the algorithm.

In Appendix A of the Physica D paper we presented Fortran code for estimating a complete Lyapunov Spectrum from a set of ordinary differential equations. That code, which has no relevance to experimental data, is not the subject of this document or the new Matlab code.

In Appendix B of the Physica D paper we included Fortran code for estimating the dominant Lyapunov exponent from an experimental time series. That code, while concise enough for publication, was not intended for "production" use, especially for large data sets, or for systems that required a relatively high embedding dimension. As we noted in Physica 16D, "we can also provide a highly efficient database management algorithm that can be used … to eliminate the expensive process of exhaustive search for nearest neighbors." Over the years, many have used the code that appeared in Appendix B, and their data sets were small enough, or their computational resources large enough, that they did not require the more efficient code. Others requested the more efficient code from us, and modified it for their own purposes or needs, most commonly making minor adjustments for their local graphics hardware.

Regardless of the language in which it has been implemented, the algorithm has been presented as two components, Basgen (dataBASe GENerator) and FET (Fixed Evolution Time).

BASGEN takes a 1-D time series as input and produces a database that will be used by FET to speed the process of finding locating neighboring points in a time-delay reconstructed data set.

FET takes as input the same 1D time series and the database produced by BASGEN and estimates the dominant Lyapunov exponent by monitoring the rate of divergence of nearby trajectories in the reconstructed phase space.

It is sensible to separate database creation from exponent estimation, as one is likely to use BASGEN once for a particular time series, then use the resulting database repeatedly in FET for different choices of FET's parameters, such as the value of time delay in time-delay reconstruction.

[1] Alan Wolf, Jack B. Swift, Harry L. Swinney, John A. Vastano, Determining Lyapunov Exponents From A Time Series, Physica 16D, p.285-317 (1985).

## Chaos and Lyapunov Exponents

Loosely speaking, "chaos" refers to deterministic (non-random) and non-periodic behavior which also appears erratic, wild, or "unpredictable." A time series that appears erratic might be chaotic, or it might be multiply periodic, periodic with "noise," or some form of pure noise. It can be very difficult to determine the source of erratic behavior in a time series, but it may also be of great importance in determining a research methodology. If a process is known to be purely noise, a statistical approach may be best. If a process is known to be non-chaotic with noise, you might try to filter out the noise and use deterministic linear modeling for the underlying dynamics. If a process is chaotic, non-linear modeling is suggested.

A defining feature of chaos is "Sensitive Dependence upon Initial Conditions" or SDIC. SDIC means that small changes in the state of a system will grow at an exponential rate and will dominate the behavior. Since an error bar is composed of many adjacent states in the solution space (or "phase space"), and since adjacent states diverge quickly, it follows that error bars on the initial conditions of chaotic systems grow exponentially fast. Error bars on initial conditions are omnipresent, so we conclude that long term predictions of chaotic systems are futile, no matter how system prediction is implemented (e.g., digital computer, analog computer, experimental realization).

SDIC is quantified with Lyapunov exponents. These numbers are the long time average exponential rates of divergence of nearby states. If a system has at least one positive Lyapunov exponent, then the system is chaotic. The larger the positive exponent, the more chaotic the system; that is, the shorter the time scale of system predictability. A system may possess any number of Lyapunov exponents, but to confirm chaos we only need to confirm that the dominant (largest, or most positive) exponent is positive. Hence estimation of the dominant exponent is especially important.

The program FET (and its preprocessor BASGEN) allows one to estimate the dominant Lyapunov exponent in any time series (a list of numbers consisting of samples of a single system variable). The program was described in Physica 16D (ref. 1) in 1985. As I stated in that paper, the FORTRAN code in Appendix B was for illustrative purposes only -- it was too inefficient for actual implementation. I have been distributing the source code to the more efficient version of the program by mail since 1985.

This document is the first official program documentation for FET/BASGEN, but it does not contain an extensive tutorial on Lyapunov exponents. For background on the subject, see references 1-7 in the bibliography at the end of this document.

## Time delay reconstruction

In studying sensitive dependence, FET and BASGEN make use of the method of time delay reconstruction (also referred to as delay reconstruction and phase space reconstruction). The method is briefly discussed in this section, however readers unfamiliar with the technique are advised to see references 12 and 13.

Delay reconstruction builds an ndim-dimensional "orbit" out of a time series once the user selects two parameters: the embedding dimension "ndim," and the time delay "tau".

Example: If ndim is chosen as 3, tau is chosen as 5, and the time series consists of the values: $x_1$, $x_2$, $x_3$ .... then the delay reconstructed orbit would consist of the following sequence of points in 3-space:

$(x_1, x_6, x_{11})$, $(x_2, x_7, x_{12})$, $(x_3, x_8, x_{13})$ ....

That is, delay reconstruction produces the ndim-tuples:

(x(t), x(t+tau), x(t+2*tau),..., x(t+(ndim-1)*tau))

An important consequence of the delay procedure is that the size (linear extent) of the reconstructed orbit in each of the ndim dimensions is equal to the difference between the largest and smallest values in the original time series.

The graphical result of delay reconstruction may be pictured as taking a single long strand of spaghetti (the time series) and twirling it in a dish so it defines a path through a bounded region of space. In twirling the strand, segments of the time series that were quite far apart (temporally) may be brought into close proximity (spatially).

Some of the important properties of delay reconstruction are:

1) Periodic time series become closed phase space orbits.
2) An infinitely long time series of white noise would become a "scatter plot" that would completely fill ndim space.
3) Chaotic time series reconstruct into fractal orbits whose orbital segments exhibit sensitive dependence.

In performing the reconstruction, the user chooses the value of ndim by the method of "educated guess" or "wild guess" to ensure that the orbit is topologically reasonable in ndim dimensions. For example, doubly periodic motion created from samples of x(t) = 3sin(5t) + 2sin(2t) will delay reconstruct into a two-torus (donut) that is well behaved in a space of three dimensions or higher. In two dimensions the orbit has an unnecessary self-intersection.

An optimal choice of tau will "fatten" the delay reconstructed orbit so that it has as simple an appearance as possible. The simpler the structure, the more accurately FET can quantify orbital divergence. The reader will be reassured to learn that tau dependence of Lyapunov exponent estimates is almost always very weak. often tau is chosen to be the number of data points that correspond to $^1/_3$rd of the mean period of the reconstructed orbit.

## Introduction to FET & BASGEN

FET (Fixed Evolution Time)

FET estimates the dominant (largest positive) Lyapunov exponent in your time series. This is accomplished by averaging the exponential rate of divergence of short segments of the delay reconstructed orbit. A much simplified outline of the process follows but does not replace a careful reading of reference 1.

1) FET creates a multi-dimensional phase space orbit from a one-dimensional time series by delay reconstruction.

2) Using the database created by BASGEN (see the next section), FET locates a pair of points that are very close to each other in the reconstructed phase space orbit.

3) FET follows each of the points as they travel a short distance along the phase space orbit. We can compare the initial separation (ordinary Euclidean distance) of these points to their separation at the end of the interval. The logarithm (base 2) of the ratio of final to initial separation of these points is a local estimate of orbital divergence.

4) If the two points are still fairly close together at the end of this interval, we keep both of them, evolve them a bit farther along the orbit, and compute the next local value of orbital divergence. If the points have grown much farther apart, we keep one of the points, and use the database to find an appropriate replacement for the other point.

5) By averaging the local rates of orbital divergence and dividing by the total travel time along the orbit we obtain the long time average rate of divergence of nearby orbits. The word nearby is important - our contributions come from orbital segments that are reasonably close together at all times.

FET presents its running estimate of the dominant Lyapunov exponent numerically at the top of the screen and also writes it to the file "fet.out". Segments of orbital divergence are displayed graphically during the calculation as a check on its robustness. This document contains graphics dumps showing the appearance of "good" and "bad" segments.

## Introduction to FET & BASGEN

BASGEN (dataBASe GENerator)

The function of BASGEN is to create a database from the user provided time series so that FET can use the time series more efficiently. With the original time series and the database, FET can quickly locate all of the points that are close to any specified point in a reconstructed phase space. While a rudimentary understanding of BASGEN is necessary to realize large performance gains in FET, users need not understand the detailed operation of the database algorithm.

BASGEN puts the reconstructed orbit points in an ndim-dimensional grid with ires boxes per side. Of the iresn$^{th}$m possible occupied boxes, the vast majority are likely to be empty, so BASGEN uses a virtual array to define the grid -- only boxes containing one or more data points consume memory. ires is user chosen so that the average occupied box contains a reasonable number of points, say 50. When FET needs the points close to a specified point in phase space, it determines which box the point is in, and looks through the database. The database returns a list of all of the points in the same box, given as the positions of their first coordinate in the original time series. FET will now work with the "50" points in that box, rather than the thousands of points in the time series.

The Lyapunov exponent algorithm in Appendix B of reference 1 did an exhaustive search for nearby neighbors instead of using a database, and runs 10 to 1000 times slower (depending on the length and other properties of the time series) than this version. Database generation could have been incorporated into FET, however most users do many FET runs (checking exponent stability) for a given database.

BASGEN reads in the original time series from the ASCII file "data." with one integer or floating point value per line. It creates a database file called "database." and a new time series file called "t.1". These are written (FORTRAN version) as "unformatted" files which are read in much more quickly than ASCII files. FET uses both of these files, but BASGEN can also read "t.1" if a new database is to be made from the same time series.

## Quick Start

The following parameter values are suggested for those who must run their own time series RIGHT NOW. More patient individuals will run the sample files with the suggested parameter values first. I assume a time series with ROUGHLY a dozen data points per mean orbital period.

### BASGEN

| Prompt | Your response |
|---|---|
| ASCII data file = 1 | 1 |
| number of data points (<= 32000) | (# of pts in data file) |
| time delay (samples) | (# pts in 1/3 orbit time) |
| embedding dimension (<= 8) | 4 |
| grid resolution (maxbox = 6000) | 8 |
| ok=l | 1 |

### FET

| Prompt | Your response |
|---|---|
| time-step (seconds or iterations) : | (data sampling rate) |
| evolution time (number of samples) : | (# pts in 1/5 orbit time) |
| minimum separation at replacement : | 0 for simulation data or 2% of the range of data values for experimental data |
| maximum separation at replacement : | 10-15% of the range of data values |
| maximum orientation error (degrees) : | 30 |

Notes:

In the prompt column, expressions in brackets such as 32000) express constraints, not default values.

In the response column, text descriptions in brackets are replaced with your own numerical values.

Where a response refers to the "range of data values" you should take compute the difference between the largest and smallest values in the time series.

The time delay and evolution time parameters refer to fractions of the "orbit time". This is the characteristic time scale for your time series -- the average duration of a wiggle. While not well defined for a non-periodic time series, this parameter does not strongly affect exponent estimation. one approach to estimating this value is to select the frequency (1/time scale) that corresponds to a dominant power spectral feature.

**More on BASGEN**

ASCII data file = 1 :

BASGEN wants to know if the time series is to be read from the original ASCII file "data." or from the unformatted file "t.1" that was created by a previous run of BASGEN. For a first run, enter 1.

number of data points (<= 32000) :

The number of points to use from the time series file. The maximum number of points may vary from 32,000 depending on local modification to the source code. As discussed in reference 1, the number of points required for a system of fractal dimension d is ROUGHLY $30^d$. These points should span ROUGHLY $10^{d-1}$ "orbits." Emphasis on ROUGHLY, I simply want to discourage the use of 2000 points for a six-dimensional system that would probably require about a billion data points ($30^6$).

time delay (samples) : (tau)

As described earlier, time delay reconstruction defines the tuples x(i), x(i+tau), x(i+2*tau), etc. (ndim times) from your time series, x(i). Lyapunov exponents show little tau dependence, so little thought is usually given to tau selection. A "convention" is to pick tau to be ROUGHLY one third of the number of points in the mean period of the motion. Visually, our goal is to select a tau that will "open up" the reconstructed orbit, so that distinct parts of the structure will appear in distinct parts of phase space. The sample segments of orbital divergence shown later in this document show the "plump" structure characteristic of a good choice of tau (see also ref. 1, figure 5).

embedding dimension (<= 8) : (ndim)

The reconstructed phase space consists of points in ndim space. This number should not be so small that the reconstruction is topologically incorrect (a 2-torus (donut) likes to sit in at least a 3-D space). Memory constraints are the biggest problem with picking a value for ndim that is larger than the minimum acceptable value. In practice, its unlikely that you will have enough data to run a system that requires an embedding dimension higher than 4, so this is a good value to start with. In the unlikely event that you have a reliable value for the fractal dimension of your delay reconstructed phase space orbit (these calculations are notoriously unstable), you might choose ndim as the fractal dimension rounded up to the next highest integer.

grid resolution (maxbox = 6000) : (ires)

BASGEN places the delay reconstructed data in a grid of dimension ndim, with a resolution of ires cells per side. Typical values for ires are in the range of 6 to 12. For ndim=5 and ires=12, a theoretical maximum of $12^5$ = 248832 cells might be required to contain the data points. As this number is probably far larger than the number of data points, it would be very wasteful to store the points in an ordinary array. Inst6ad, a "virtual grid" is used, which only requires storage for boxes that actually contain data points. When you enter this parameter, BASGEN reports on the number of (non-empty) boxes it used to partition the orbit.

When FET needs the phase space points near a given phase space point, it determines which box contains the specified point, looks through the BASGEN generated database to determine which other

points are in that box (if any) and works with those points. If there are no suitable points in the same box, FET examines the grid to see if the nearest neighbor boxes contain suitable points, and so on. In a 3-D reconstruction each box has 26 ($3^3$-$1^3$) nearest neighbor boxes, 98 ($5^3$-$3^3$) next nearest neighbor boxes, and so on.

Three points about ires selection should be made:

–If ires is too small, cells will tend to contain too many points that are not all close to each other. The efficiency of the database approach is lost.

- If ires is too large, many boxes will contain a single data point. Many layers of boxes will have to be examined to find suitable neighbors.

–Within reasonable bounds, the choice of ires should affect the efficiency, but not the accuracy of the exponent estimate.

Since we cannot tell in advance how many boxes will be required, we dimension our arrays for the virtual grid for up to "maxbox" boxes. If this number is exceeded through your choice of ires, BASGEN will ask for a new ires value and will restart the binning process.

When BASGEN has completed the binning process, it will tell you how many non-empty boxes were-re- quiredIfyo-u are satisfied, respond to-the prompt.. olc.1 by typing 1, and the database file will be written. If you are not satisfied with the number of non-empty boxes, type any other value and you can respecify ires.

For my typical time series (32k points and an ndim of 3 or 4) I find an ires of 6 to 8 appropriate. This usually results in several hundred boxes, which on the average contain ROUGHLY 100 points.

**More on FET**

time-step (seconds or iterations) :

This is the time between samples in the time series. If you enter 1 for this parameter, the Lyapunov exponent will have the units of bits per data point (or bits per iteration for a discrete mapping). other values for this parameter will be interpreted as the number of seconds per data point and will multiply the exponent estimate to produce the units of bits per second.

evolution time (number of samples)
(evolve)

Each pair of points will be followed through the phase space for this number of steps, at which point the local contribution to orbital divergence is computed, and a replacement is attempted if necessary. FET's graphical display shows the path of the two points through phase space and the distance between the points at each step as a "rung" on a ladder. See the section "Interpreting graphical and text output" for examples of this display with an evolve of about 6.

evolve should be kept small enough that orbital divergence is monitored at least a few times per orbit, and sensitive dependence does not pull the points too far apart. (Strictly speaking, Lyapunov exponents quantify SDIC for orbital segments whose separation, though growing quickly, always remains infinitesimal.)

If you had 12 data points per "mean orbital period" you might choose evolve to be 3 (or 4) so that orbital divergence was checked 4 (or 3) times per orbit. If your goal is a convincing graphical image of sensitive dependence in your system rather than a stable Lyapunov exponent estimate, you may want to increase evolve by a factor of 2 or 3 and modify the graphics so that several orbital segments are displayed between screen erasures.

You may find a fairly strong dependence of the Lyapunov exponent on evolve. If we had an infinite amount of data, we could probe orbital divergence at infinitesimal separations. since we are limited to the analysis of a finite data set we are stuck with estimates of orbital divergence on length scales that range from the average starting separation between pairs of points (on the low end) to the average distance between these points after evolve steps (on the high end).

THERE IS NO GUARANTEE THAT ORBITAL DIVERGENCE ON A RANGE OF FINITE LENGTH SCALES IS QUANTIFIED BY THE "TRUE" LYAPUNOV EXPONENT (FOR INFINITESIMAL LENGTH SCALES).

minimum separation at replacement : 0 to 2% of range of time series values (dismin)

If the separation between diverging phase space points exceeds dismax (the next parameter) FET attempts a replacement for one of the points. Since the location of each phase space point has an error bar, a replacement point that is <u>very</u> close to the "kept" point may define a new initial separation with a large relative error. This is most likely to occur for very long and/or noisy time series. To reduce this effect we reject replacement points whose distance from the kept point is less than dismin. For very high accuracy time series this parameter may be set to zero or made very small. For experimental data with little "noise," dismin should be set to a few percent of the range of time series values.

dismin is the smallest length scale on which orbital divergence is monitored, so it may significant affect your exponent estimate. Try both zero and non-zero values.

maximum separation at replacement : 10% to 15% of range of time series values (dismax)

dismax determines whether two orbital segments will be followed for another evolve time steps or a replacement will be attempted. The parameter defines the largest length scale on which orbital divergence is being monitored, i.e., the largest length scale that (hopefully) behaves similarly to infinitesimal length scales.

Recall that a reconstructed orbit has a linear extent in each dimension equal to the range of time series values. This means that the greatest possible distance between two diverging points in the orbit is a factor of ndim" larger (diagonal of a unit square is $2^{5}$, diagonal of a unit cube is $3^{05}$, etc.).

For 3-D or 4-D reconstructions I have generally found a dismax of 10-15% of the range of time series values to be optimal. If I had a time series of great length I might decrease this value to perhaps 5% of the range. For larger ndim I would increase dismax from 15% by factors of (ndim/3)" to account for the larger diagonal extent of the orbit. (dismin should also be altered in this manner.)

The need to choose dismin and dismax may be considered a feature rather than a liability, as we can focus on orbital divergence on length scales of particular interest to us. For example, in a fluid system we might choose to monitor sensitive dependence in a large scale feature of the flow.

maximum orientation error : 30 (degrees) (thmax)

During the replacement process, FET attempts to preserve the orientation of the line segment between the two points that had just been followed. thmax is the largest allowed angular deviation from an identical orientation. Preserving the orientation of diverging orbital segments is crucial if the local divergence rates are to be correctly averaged into a global Lyapunov exponent. While 30° always seems to work well, I did not want to hide this value in the FET source code.

**Running the sample files**

Three sample files have been provided so you can...
–verify program operation (especially if you modify and recompile the programs). - see what good text and graphical output looks like.
–start with a good set of program parameters so you can see the effect of parameter variations.

To run a sample file...

1) Copy one of the three files to filename "data."
2) Run BASGEN with the suggested parameters.
3) Run FET with the suggested parameters.
4) Print or type the file "fet.out" to see the final exponent estimate (or monitor the line of text at the top of the screen during program execution).

Keystroke sequence:

| | |
|---|---|
| copy data.ld data. | [Enter] <= press the Enter key |
| basgen | [Enter] |
| 1 | [Enter] |
| 512 | [Enter] |
| 1 | [Enter] |
| 2 | [Enter] |
| 20 | [Enter] |
| 1 | [Enter] |
| fet | [Enter] |
| 1 | [Enter] |
| 3 | [Enter] |
| 0 | [Enter] |
| 0.05 | [Enter] |
| 3 0 | [Enter] |
| print fet.out | [Enter] |

Test file #1 - data.ros

File contains the x component of the solution to the Rossler attractor (3 ordinary differential equations) with parameters a=0.15, b=0.2, c=10.0 (see ref. 1, Table 1), with 4096 samples taken every 0.3 seconds (20 points per orbit).

Correct value of dominant Lyapunov exponent = 0.13 bits/sec.
FET estimate is 0.1233 bits/sec. with the parameters:

| | |
|---|---|
| BASGEN | 4096, 5, 3, 10 (makes 173 non-empty boxes) |
| FET | 0.3, 61, .0001, 4., 30. |

This is an extremely well-behaved system due to its simple phase space structure.

Test file #2 - data.lor

File contains the x component of the solution to the Lorenz attractor (3 ordinary differential equations) with parameters of b=4.0, sigma=16, r=45.92, with 8192 samples taken every 0.05 seconds (12 points per orbit).

Correct value of Lyapunov exponent = 2.2 bits/sec. FET estimate is 2.2041 bits/sec. with parameters:

| | |
|---|---|
| BASGEN | 8192, 3, 4, 10 (makes 462 non-empty boxes) |
| FET | .05, 4, 0.0001, 8., 30. |

This is a more difficult system because of its two-lobed structure. By varying FET parameters over a broad but reasonable range, you may obtain estimates with errors as large as 30 percent.

Test file #3 - data.1d

File contains 512 iterates of logistic equation, x(n+l) = 4.0 * x(n) * (1-x(n)).

Theory predicts lyapunov exponent = 1.0 bits/iteration.
FET estimate is 0.98 bits/iteration with the parameters:

| | |
|---|---|
| BASGEN | 512, 1, 2, 20 (makes 53 non-empty boxes) |
| FET | 1, 3, 0.0001, 0.05, 30. |

Note that the graphics will look "disjointed" for this system, as the data comes from a discrete map rather than a continuous system.

Make up your own test data set with periodic data (with and without a few percent of added noise) and confirm that the dominant exponent is close to zero -- hence no chaos. All time series files should contain ASCII data values, one per line, either integer or floating point.

## Interpreting graphical and text output

An excellent segment of orbital divergence. The two orbital points start very close together (upper left) and remain close through evolve time steps. Most of your segments should look this good. Actually, the projection effect (3-D to 2-D in this case) could be misleading us -- the points could be far apart perpendicular to the paper, however the appearance of the previous and subsequent segments suggested otherwise. Since initial and final separations cannot easily be estimated from the figure, we can't tell how large a contribution this segment will make to the long time average Lyapunov exponent.

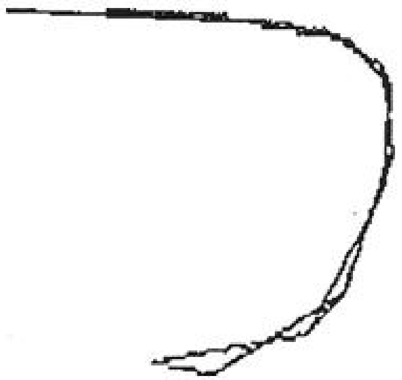

A marginal segment of orbital divergence. The points started fairly far apart and remained far apart (once again, previous and subsequent segments suggested an insignificant projection effect). The initial and final separation were each about 10% of the extent of the reconstructed orbit, larger than we generally like to see. If such segments occur often you may want to: use a longer time series, reduce mindis, reduce maxdis, or reduce evolve.

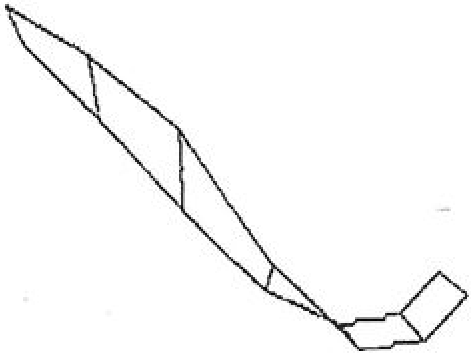

A bad segment. Two points that were reasonably close together diverged very quickly to 50% of the extent of the reconstructed orbit. We hope to do replacements before divergence becomes so "global." If more than 1 out of a 100 segments looked this bad I'd try some of the remedies listed above for the marginal segment.

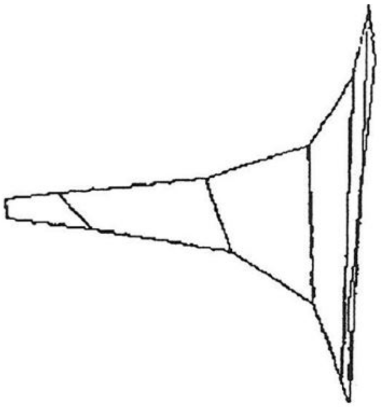

**Interpreting graphical and text output**

FET file output is exactly the same as the text output on the screen during program execution-The first column is the current location within the reconstructed orbit, which increases in steps of evolve. The second and third columns are distance between the two points at the beginning and end of the current segment. The fourth column is the running estimate of the Lyapunov exponent. The 5th column contains the change in angular orientation (in degrees) at a replacement.

Below, a section from a good run. Few replacements were necessary, and those involved a small change in orientation (6, 10, and 9 degrees). The most severe divergence occurred prompted the second replacement – two points that were 1.58 distance units apart diverged to 3.9 units apart (this was about 15% of the extent of the reconstructed orbit). The replacement brought the two points back to a distance of 0.94 units apart.

| | | | | |
|---|---|---|---|---|
| 70 | 2.1737 | 2.4943 | 0.0775 | |
| 75 | 2.4943 | 3.4036 | 0.0783 | |
| 80 | 2.3704 | 1.5814 | 0.0661 | 6 |
| 85 | 1.5814 | 3.9293 | 0.0776 | |
| 90 | 0.9487 | 0.7066 | 0.0686 | 10 |
| 95 | 0.7066 | 0.8768 | 0.0683 | |
| 100 | 0.8788 | 1.3740 | 0.0713 | |
| 105 | 1.3740 | 0.9938 | 0.0635 | |
| 110 | 0.9938 | 1.8053 | 0.0684 | |
| 115 | 1.8053 | 1.8424 | 0.0657 | |
| 120 | 1.8424 | 2.1883 | 0.0650 | |
| 125 | 2.1883 | 2.3591 | 0.0633 | |

Below, a segment of a bad run. Too many replacements, many at large angles. Much dramatic orbital divergence – 27 distance units corresponds to about 90% of the reconstructed orbit's extent!

| | | | | |
|---|---|---|---|---|
| 75 | 3.2175 | 3.9841 | 0.1283 | 17 |
| 80 | 3.9841 | 5.1626 | 0.1249 | |
| 85 | 3.8006 | 6.0229 | 0.1254 | 19 |
| 90 | 4.3086 | 13.9831 | 0.1373 | 2 |
| 95 | 3.9591 | 2.9490 | 0.1256 | 8 |
| 100 | 2.9490 | 1.7449 | 0.1117 | |
| 105 | 1.7449 | 5.7249 | 0.1228 | |
| 110 | 2.9232 | 2.4785 | 0.1150 | 25 |
| 115 | 2.4785 | 4.3921 | 0.1172 | |
| 120 | 4.3921 | 4.8711 | 0.1135 | |
| 125 | 4.8711 | 27.5141 | 0 1290 | |
| 130 | 4.3102 | 20.0489 | 0.1411 | 22 |
| 135 | 4.6337 | 10.6694 | 0.1448 | 27 |
| 140 | 3.7029 | 7.4187 | 0.1468 | 22 |
| 145 | 4.4100 | 14.0373 | 0.1532 | 18 |

**The Ideal Time Series**

If you have read the earlier sections of this document you should be able to appreciate the properties of an ideal time series:

1) A total time series length that spans at least several hundred "orbital periods".

FET averages local rates of orbital divergence to produce a long time average. The more orbits followed, the more stable the average. Furthermore, with additional orbits of data we can find pairs of points that start closer together and stay closer together for a given period of time.

Before using FET <u>always</u> plot out the full length of your entire time series. **If you see something similar to the figure below, do not waste your time attempting to extract a dominant exponent!** This time series shows both a large number of high frequency oscillations, which is good, but also one or two cycles of a large amplitude low frequency oscillation. With just a few cycles of this low frequency motion, the data set does not repetitively sample each region of phase space so an accurate average rate of phase space divergence cannot possibly be obtained.

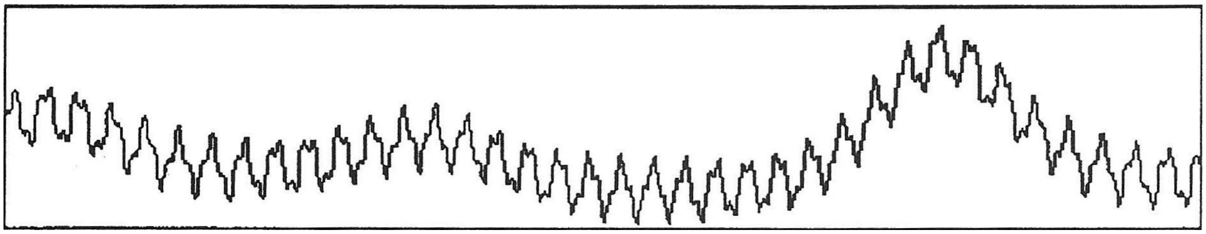

2) A sampling rate that provides ROUGHLY a dozen data points per mean orbital period.

Your eye might prefer more points per orbit to get a better sense of orbital continuity, but FET only needs to monitor orbital divergence a few times per orbital period. More points per orbit is not generally a problem (though see item 3 below), but you might increase the run time substantially and unnecessarily. We never want more points per orbit if the tradeoff is fewer orbital periods of data. As few as 4 - 6 points per orbit may be okay (tau and evolve would be 1 or 2), but this is pushing our ability to monitor the <u>gradual</u> divergence of phase space orbits.

3) As small a noise level as possible.

Each segment of orbital divergence contributes log(fnal separation/initial separation) / duration of segment to the dominant Lyapunov exponent. Noise in a time series jostles the location of points in the reconstructed phase orbit, interfering with distance ratios. This problem actually grows worse as the length of your time series increases„ because the average distance between phase space points may decrease to the same size as the location error!

In some cases, low pass filtering experimental data may alter the value of the estimated Lyapunov exponent, see reference 14, so be careful with any type of filtering or averaging. If raw data and filtered data give similar results, I'd consider the exponent estimate robust. If the filtered data has an exponent estimate that changes continuously and significantly with the choice of filter cutoff frequency, I'd be suspicious of the filtered results.

**Interpreting the exponent estimate**

If FET estimates the dominant Lyapunov exponent as p bits per second, and if your time series data contain q "good" bits, you will lose p of the q bits of information each second, so that complete predictability is lost after approximately q/p seconds.

Example

Your data consists of 12-bit temperature values as a function of time. It is known that the temperature sensor itself is only accurate to 8 bits, even though the digital circuitry gives you 12-bits of data (the lowest 4 bits are "noise"). For the purpose of estimating information loss we consider that you begin with 8 good bits of data.

Repeated runs of FET suggest that the dominant Lyapunov exponent is about +0.5 bits/second. You will be losing about 0.5 bits of your 8.0 bits of initial information each second - on the average. You will lose predictability - on the average - after about 8.0/0.5 = 16 seconds.

The same result may be obtained somewhat differently. Given 8-bit data, your initial error bar was 1 part out of $2^8$ = 256, or about 0.4%. The error bar was growing as $(0.4) \cdot 2^{(0.5\ t)}$. Once this value reaches 100.0%, at t = 16 seconds, you'd have lost all predictive power.

That is, in 16 seconds, the various initial conditions in your error bar could have evolved to virtually any other possible state in the system. Do not forget the caveat "on the average". There are likely to be 16 second segments during which the error bar shrunk, stayed the same in size, or grew at various rates. we can only be sure that the average time series segment of this duration would show the orbital divergence computed above.

If the estimated exponent is very large your time series may contain more noise than chaotic signal. In a time series of pure white noise, any two points that delay reconstruct to be close together at one time are likely to be very far apart at the next time step. The graphical "ladder" of orbital divergence (see the preceding section) will show explosive rather than gradual growth. An additional indication of suspicious orbital divergence is very frequent replacements (especially at large angles) as noted in the file "fet.out".

If the estimated exponent is near zero the system is exhibiting some sort of orbital stability, or periodicity.

When is an estimated exponent considered small? very small? A simple example will demonstrate that the absolute size of the exponent in bits per second cannot answer this question. Consider a nearly perfectly periodic data file consisting of 32,000 points spanning 1000 orbits. Not a single replacement is necessary during the entire run of FET, so the Lyapunov exponent will consists of the single contribution: log(final distance/initial distance) / total-time.

Suppose that (final distance/initial distance) equals 2, which is very little divergence for 1000 orbits. The exponent estimate in bits/second will then be: 1 / total-time.

Depending on how long the system takes to complete its 1000 orbits, the Lyapunov exponent for this non-chaotic system could take on any positive value whatsoever.

For the purpose of evaluating an isolated estimate of the dominant exponent, convert the exponent to units of bits/orbit by multiplying the FET output value (in bits/second) by a ROUGH estimate of the number of seconds per "orbit". In the above example, the Lyapunov exponent would be +0.001 bits/orbit, which is small enough to be considered indistinguishable from 0.

If you are studying a system that makes a transition from periodicity to chaos, the question "How big is my exponent?" is less problematic, as you will hopefully find that the exponent grows by a few orders of magnitude, clarifying which values are "near zero" (see ref. 7).

## Comments, Bug Fixes, Enhancements

BASGEN and FET have not been significantly changed since I first started to distribute code in 1985. Some of the following comments are bug fixes, all of which were implemented long ago in the distribution disk.

A few of the comments that follow are now historical curiosities, as personal computers now routinely have 64 bit operating systems and large amounts of inexpensive RAM.

### Bug

There was a bug in some previously distributed copies of FET FORTRAN source and executable (I can't be precise about the dates of distribution of the bad code). The array newcrd in subroutine "search" was intended to be of type real, but defaults in FORTRAN to type integer. This will only be important if integerizing your data set significantly alters the data values (e.g.. irrelevant if your data values are large integers).
The fix is to replace all occurrences of newcrd with zewcrd or another variable name of type real (starting with letters a-h, o-z). Alternately, declare variable newcrd explicitly as real.

### Bug

In the FORTRAN source for BASGEN I had intended to symmetrically enlarge the phase space grid so all data points would fall inside the grid, rather than on the boundary, but I accidentally introduced a small bias. Technically this is a bug, but it should have no significant effect on the estimated exponent. For the sake of consistency ...

old code :

```
datmin = datmin - 0.01 * (datmax-datmin)
datmax = datmax + 0.01 * (datmax-datmin)
```

Note that datmin is not decreased by the same amount that datmax is increased, as datmin has already been changed when it is used in the second line.

new code :

```
datdif= 0.01 * (datmax-datmin)
datmin = datmin - datdif
datmax = datmax + datdif
```

### Bug

Both BASGEN and FET had poor file handling code that forced users to delete old output files (such as fet.out) at the DOS prompt prior to new runs. New code with the Inquire statement (FORTRAN 77) fixes this.

### Bug/Enhancement

When the final distance between a pair of "diverging" orbital segments happened to be zero, FET would crash as it attempted to compute log (final distance / initial distance) = log (0). This problem arises VERY infrequently, usually with experimental data of very low resolution (say, 6 bits). The fix is not a sophisticated one, we simply ensure that the contribution from this orbital segment will not dominate the average exponent.

added FORTRAN : if (disnew. lt.0.1*dismin) disnew=0.1*dismin
This line appears as follows...

```
80          continue
            disnew=sqrt(disnew)
            if (disnew.lt.0.1*dismin) disnew=0.1*dismin
```

added C : if (disnew<0.1*dismin) disnew=0.1*dismin;

This line appears as follows...

```
disnew = (float)sqrt((double)disnew);
if (disnew<0.1*dismin) disnew=0.1*dismin;
```

## Possible bug

Be careful with any recoding of the call to the logarithm function in FET. There has been confusion in the literature about the values of Lyapunov exponents of some well-known systems. Values often disagree by factors of the logarithm of 2, e, or 10 in another base. ALWAYS use log base 2 to obtain the Lyapunov exponent in the preferred units of bits per time unit. ("Preferred" so that the time for which you may predict the state of your system can be determined from: [initial information about the system in bits] – [rate of information loss in bits/sec]*[time in seconds] = 0.)

To ensure that this is done correctly, simply compute...

```
log(x)/log(2.0)
```

This will be correct for any base of the local log function.

## Memory Requirements

The FORTRAN source (and the PC executables) on the distribution disk contain the parameters:

maxdat = 32000, maxdim = 8, maxbox = 6000

where maxdat refers to the maximum length of the time series, maxdim refers to the maximum embedding dimension of the time series, and maxbox refers to the maximum number of boxes (grid elements) required to partition the data in an ndim-dimensional virtual grid of resolution ires. It is theoretically possible that $iresn^{di}m$ boxes would be required to partition the data set, but in practice this value is usually far smaller.

These values may be changed in the parameter statement:

```
parameter(maxdat=3 2000,maxbox=6000,maxdim=8)
```

Note that the parameter statement appears once in BASGEN and twice in FET (once in the main program and once in the search subroutine).

The total amount of dimensioned array elements is approximately the same in BASGEN and FET:

| | |
|---|---|
| real | maxdat |
| integer*2 | 2*maxbox*maxdim + maxdat + maxbox |

For 4-byte (32 bit) reals and 2-byte (16 bit) integers, this totals about 400,000 bytes of array space for my default values of the 3 parameters. If you are having memory problems simply reduce the parameters values to:

maxdat = 16000, maxbox = 4000, maxdim = 4

which requires about 200,000 bytes of storage and will be adequate to run the test data files.

## Memory Requirements and > 32,000 Data Points

If you wish to run more than 32,000 data points, the easiest modification to make to both FORTRAN programs is to simply change all integer*2 variables to integer*4 variables. Be sure to do this in the internal subroutines as well as the main programs.

## Parameter name changes

| | |
|---|---|
| FET/BASGEN code -- | EVOLVE, DISMIN, DISMAX, THMAX |
| Physica 16D code -- | EVOLV, SCALMN, SCALMX, ANGLMX |

## Convergence

Slow convergence is typical for Hamiltonian (non-dissipative) systems. These systems contain no attractors, so are generally of higher phase space dimension. The result is that a stable average will require many more local divergence rates. The dependence of time series length on dimension is exponential, so increased phase space dimension can be a big problem. No quick fix is possible in this case. Try to obtain and run much longer time series.

## FET graphics

It is strongly recommended that FET be implemented with the local graphics calls necessary to enable the display of diverging orbital segments. The FORTRAN executable on the distribution disk was produced with a commercial graphics library (GRAFEX) with two calls ...

qline(xl,y1,x2,y2,1) -- which draws a line from (xl,yl) to (x2,y2) in color 1.

qcmov(col,row) -- which moves the invisible text cursor in graphics mode so the running estimate of the exponent is printed on the top line of the display.

In FET.FOR I have isolated most of the graphics calls (except for a qcmov or two) to a section surrounded by "********". This section can be commented out for no graphics, or modified to meet local graphics requirements.

## FORTRAN compiler directives

The first three lines of FORTRAN source for FET and BASGEN are compiler directives for the Microsoft (IBM PC) FORTRAN compiler ver. 4.01. For other PC or mainframe compilers this code should be removed.

## C version

Joe Mietus was kind enough to transcribe the FORTRAN versions of FET and BASGEN to C. This was a literal line by line code translation, so the resulting code is not optimized for C. We verified that to machine precision C and FORTRAN codes generated identical intermediate results as well as identical exponent estimates for a test file.

I have since compiled and run the C version on several PC, mini, and mainframe computers and have had no problems -- with the exception of Turbo C. This compiler had problems with memory consumption (even in huge mode) and file i/o. Since I am not a serious C programmer, please contact your local C guru first if you have implementation problems.

## Code variation

I toyed with the idea of "hardwiring" certain parameters into the code, such as fixing thmax at 30 degrees. In the end, I decided not to "hide" these parameters.

A few copies of code were distributed with hardwired parameters along the lines suggested in the "Quick Start" section:

dismin = 0.02 * data range (range = difference between largest and smallest time series value)
dismax = 0.15 * data range
thmax = 30 degrees

## A comment on matrix techniques for estimating Lyapunov exponents from experimental data

There have been several attempts to estimate additional Lyapunov exponents from experimental data sets by estimating local Jacobian matrices, taking the product of many of these, and extracting all of the eigenvalues. These methods are an obvious and well-intentioned extension of the approach of monitoring the dominant contribution to orbital divergence, but I believe that they have several problems that make them unworkable for real data sets:

1) Additional exponents will be less stable than the already difficult to determine dominant exponent.

2) Matrix multiplication compromises even the stability of the largest exponent estimate, due to mixing of some very poorly determined local divergence rates with some well determined rates.

3) In at least one numerical study (ref 6), such a method produced spurious large positive exponents. This is as severe a failing of exponent estimation as one could make.

**BIBLIOGRAPHY**

Articles about the numerical estimation of the dominant Lyapunov exponent:


1) Determining Lyapunov Exponents From A Time Series
Alan Wolf, Jack B. Swift, Harry L. Swinney, John A. Vastano
Physica 16D, p.285-317 (1985).

2) Quantifying Chaos with Lyapunov Exponents
A. Wolf
Chapter 13 of Nonlinear Science: Theory and Applications, ed. Arun Holden,
Manchester University Press (1986).

3) Diagnosing Chaos in the Space Circle
Alan Wolf and Tom Bessoir
Physica 50D, p.239-258 (1991).

4) Intermediate Length Scale Effects in Lyapunov Exponent Estimation
A. Wolf and J.A. Vastano
Dimensions and Entropies in Chaotic Systems, Ed. G. Mayer-Kress,
Springer-Verlag (1986).

5) Progress in Computing Lyapunov Exponents from Experimental Data ** This paper is now obsolete **
A. Wolf and J. Swift
in Statistical Physics and Chaos in Fusion Plasmas, ed. W. Horton,
Wiley Interscience, New York (1983).

6) Comparison of Algorithms for Determining Lyapunov Exponents from Time Series
J.A. Vastano and E.J. Kostelich
Dimensions and Entropies in Chaotic Systems, ed. G. Mayer-Kress, Springer-Verlag (1986).

7) Low-Dimensional Chaos in a Hydrodynamic System
A. Brandstater, J. Swift, H.L. Swinney, A. Wolf, J.D. Farmer, E. Jen, J.P. Crutchfield
Phys. Rev. Lett. 51, p.1442 (1983).


Also on the subject of Lyapunov exponents:


8) Lyapunov characteristic exponents for smooth dynamical systems and for Hamiltonian systems; a method for computing all of them.
Bennetin, G., Galgani, L. and Strelcyn, J.-M.
Meccanica 15, p.9-20 (1980).

9) Probleme general de la stabile du movement
Lyapunov, A.M.
Ann. Math. Study 17 (1947).

10) A multiplicative ergodic theorem. Lyapunov characteristic numbers for dynamical systems.
Osledec, V.I.
Trans. Moscow Math. Soc. 19, p.197 (1968).

11) A numerical approach to ergodic problem of dissipative dynamical systems.
Shimada, I. and Nagashima, T.
Prog. Theor. Phys. 61, p.1605-16 (1979).

On delay reconstruction:

12) Geometry from a Time Series
N. Packard, J.P. Crutchfield, J.D. Farmer, and R.S. Shaw,
Phys. Rev. Lett. 45(9), p.712 (1980).

13) Dynamical Systems and Turbulence, Warwick, 1980 Lecture Notes in Mathematics, 898
F. Takens
Eds. D.A. Rand and L.-S. Young, Springer (1981).

On numerical problems relating to low pass filtering:

14) On the Fractal Dimension of Filtered Chaotic Signals
R. Badii and A. Politi
Dimensions and Entropies in Chaotic Systems, Ed. G. Mayer-Kress, Springer-Verlag (1986).
